# Effects of rumen cannulation combined with different pre-weaning feeding intensities on the intestinal, splenic and thymic immune system in heifer calves several month after surgery

**DOI:** 10.3389/fimmu.2023.1160935

**Published:** 2023-04-18

**Authors:** Wendy Liermann, Lisa-Maria Tümmler, Björn Kuhla, Torsten Viergutz, Harald Michael Hammon

**Affiliations:** ^1^ Institute of Nutritional Physiology "Oskar Kellner", Research Institute for Farm Animal Biology (FBN), Dummerstorf, Germany; ^2^ Institute of Reproductive Biology, Research Institute for Farm Animal Biology (FBN), Dummerstorf, Germany

**Keywords:** milk replacer, feeding regime, T and B cell, mesenteric lymph node, intraepithelial lymphocyte (IEL)

## Abstract

Fistulation is a helpful procedure in animal nutritional research and also common practise in human medicine. However, there are indications that alterations in the upper gastrointestinal tract contribute to intestinal immune modulations. The present study aimed to investigate effects of a rumen cannulation in week 3 of life on the intestinal and tissue specific immune system of 34-week old heifers. Nutrition influences the development of the neonatal intestinal immune system to a high extent. Therefore, rumen cannulation was investigated in combination with different pre-weaning milk feeding intensities (20% (20MR) vs. 10% milk replacer feeding (10MR). Heifers of 20MR without rumen cannula (NRC) showed higher cluster of differentiation (CD)8^+^ T cell subsets in mesenteric lymph nodes (MSL) compared to heifers with rumen cannula (RC) and 10MRNRC heifers. CD4^+^ T cell subsets in jejunal intraepithelial lymphocytes (IELs) were higher in 10MRNRC heifers compared to 10MRRC heifers. CD4^+^ T cell subsets in ileal IELs were lower and CD21^+^ B cell subsets were higher in NRC heifers compared to RC heifers. CD8^+^ T cell subsets in spleen tended to be lower in 20MRNRC heifers compared to all other groups. Splenic CD21^+^ B cell subsets were higher in 20MRNRC heifers compared to RC heifers. Splenic *toll like receptor* (*TLR*) *6* expression was increased and *IL4* expression tended to be increased in RC heifers than NRC heifers. Splenic *TLR2*, *3* and *10* gene expression was higher in 20MR compared to 10MR heifers. Jejunal *prostaglandin endoperoxide synthase 2* expression was higher in RC heifers than NRC heifers, and *MUC2* expression tended to increase in 20MR heifers compared to 10MR heifers. In conclusion, rumen cannulation modulated T and B cell subsets in the down streaming gastrointestinal tract and spleen. Pre-weaning feeding intensity seemed to affect intestinal mucin secretion and T and B cell subsets in MSL, spleen and thymus until several month later. Interestingly, in MSL, spleen and thymus the 10MR feeding regime evoked similar modulations of T and B cell subsets like rumen cannulation.

## Introduction

Rumen cannulation is a method allowing the investigation of physiological and biochemical conditions of the ruminal digestive tract over a longer period of time (1). Its important role in the research for new nutrient sources, control of greenhouse gas emissions or the research for effects of ruminal production systems on ruminal balance, diseases and microbiota was summarized in a review of Castillo and Hernandez (1). Also, in human medicine fistulation of the stomach or the jejunum and especially the percutaneous endoscopic gastrostomy (PEG) are used to feed or support patients with a dysfunctional gastrointestinal system in the long term (2). It is reported that fistulation or cannulation has no major effects on feed intake, growth performance, gross anatomy of the upper gastrointestinal tract or health of young calves (1, 3). Nevertheless, it was demonstrated that rumen cannulation alters the ruminal gas concentrations, methane production as well as the microbial community in cows and goats (4–6). In humans, it was shown that PEG can lead to bacterial overgrowth in the upper gastrointestinal tract and a reduction in the pro-inflammatory cytokine response of the gastric and small intestinal mucosa (7). The influences of the rumen cannula placed in the pre-weaning period on the intestinal and systemic immune system of growing calves have not been investigated so far.

The intestine is the major absorption site for nutrients of a ruminant in the pre-weaning period. Intestinal immune cells together with Peyer´s patches (PP) are the greatest immune instruments in mammals playing a key role in the defense against microbes and pathogenic factors present in the lumen of the digestive tract. The intestinal and systemic immune responses and calf health are likely influenced by the level of milk fed to calves prior to weaning (8–10). In an attempt to reduce feed costs and to accelerate ruminal development, restrictive milk feeding supporting early consumption of high amounts of solid feed have been common practice in calf rearing which has led to a decrease in weaning age. However, various studies revealed positive effects on growth performance and milk production when calves received a higher milk plane (8, 11–13). Furthermore, the provision of adequate amounts of nutrients such as amino acids, carbohydrates, as well as fatty acids is crucial for intestinal growth, maintenance of the gastrointestinal homeostasis, and the establishment of intestinal health (14). A higher plane of milk feeding seemed to have beneficial effects on the defensive power against pathogens and long lasting growth-stimulating effects on the small intestinal mucosa in calves (8, 15–17). Studies by Hammon et al. (18) demonstrated that a higher milk replacer (MR) feeding level increased the activation of upstream regulators of the intestinal mucosal immune system. The authors also found that the feeding intensity modulated T cell regulation and activation as indicated by altered gene expression of indoleamine 2,3-dioxygenase and pro-inflammatory cytokines such as interleukin (IL)-1, IL-6, and tumor necrosis factor-alpha (18). In contrast, malnutrition or total parenteral feeding can adversely affect the gastrointestinal morphology and barrier function and the development of the intestinal microbial community (19–23). Studies by Burkholder et al. (24) and Zhang et al. (25) showed that even temporal feed restriction reduces the barrier function and altered the microbial composition in the intestine.

Considering the superior role of the intestine in young ruminants and the mentioned effects on the intestinal microbiota or the intestinal immune system associated with the implantation of a fistula we hypothesized that rumen cannulation in an early stage of life influences the intestinal and systemic immune system of cattle in later life. We further hypothesized that a restrictive MR feeding regime exacerbates the impact of rumen cannulation performed in early life.

Therefore, the present study aimed to investigate the effect of pre-weaning rumen cannulation of calves fed two different MR intensities on the intestinal and systemic immune system in week 34 of life.

## Material and methods

All procedures and housing conditions of the current experiment were performed in accordance with the guidelines of the German Animal Protection Law at the experimental facilities of the Research Institute for Farm Animal Biology (FBN), Dummerstorf, and were approved by the Landesamt für Landwirtschaft, Lebensmittelsicherheit und Fischerei Mecklenburg-Vorpommern, Rostock (registration number 7221.3-1.1-009/16).

### Experimental design and animals

The experimental design was already published by Tümmler et al. (26). Briefly, 24 female Holstein calves, born at two dairy farms nearby the FBN, were separated from their dams and transported to the experimental facilities of the FBN immediately after birth. They were housed individually in pens littered with wood shavings. The calves were divided according to their body weight in two feeding groups differing in their pre-weaning milk feeding intensity (10%, 10MR versus 20% MR, 20MR feeding level relative to body weight; n = 12).

The 10MR group received colostrum from the same pool in an amount of 10% of body weight per day provided in two meals per day at the first two days of life. On d 3, the 10MR group was offered 5% of birth weight in colostrum (first meal) and 5% of birth weight in MR (second meal). Thereafter, an amount of 10% MR of body weight was offered to 10MR calves. The MR (BERGIN Milch LC 50, Bergophor Futtermittelfabrik Dr. Berger GmbH & Co. KG, Kulmbach, Germany) was fed by an automatic feeding system (Kälbermama Lifestart, Urban GmbH & Co. KG, Hude/Wüsting, Germany) in concentrations of 140 g powder/kg milk. The total amount of MR was provided in four meals per day. Animals were weighed weekly to adjust MR allowance.

The 20MR group received colostrum in an amount of 12% of birth weight per day also provided in two meals on the first two days of life. On day 3, the 20MR group was offered 7% of birth weight in colostrum (first meal) and 7% of birth weight in MR (second meal). Thereafter, the 20MR calves received daily MR in amounts of 20% of body weight according to the conditions described for the 10MR group, however, after week 6 the total amount of MR was divided into six meals per day. From week 9 to week 10, the amount of MR was gradually reduced to an amount of 10% MR of body weight.

Both groups were gradually weaned from MR between week 11 and 12 of life. From birth until week 12, a common starter feed was available *ad libitum* and from week 13 to 14 the starter was limited to 2 kg per day. From week 14 until week 16, the starter was gradually reduced. Hay was offered *ad libitum* from birth until the end of week 14. From week 11 until the end of the study all calves received a total mixed ration (TMR) for *ad libitum* intake. The calves had free access to drinking water at all times. Composition and ingredients of the TMR, starter, hay and MR are summarized in **Supplemental Tables S1**, **S2**.

At week 3 (18.4 d ± 0.8) of life, seven animals per feeding group were equipped with a permanent rumen cannula. During the surgery, the animals were treated with local anaesthetics, analgesics, sedatives and antibiotics. The treatment with analgesics and antibiotics were continued for the next 5 ± 1 days after surgery. Half of the animals were dehorned because of technical reasons using sedatives, analgesics and local anaesthetics on day 48 ± 8 of life.

The animals were slaughtered by bolt shooting and exsanguination in week 34 of life. At least 4 ± 1 days before slaughter daily TMR intake was measured and on the day of slaughter body weight of the non-fasted heifers was determined.

### Hematology

Blood samples were taken from the jugular vein on the day of slaughter and collected in tubes containing 1.6 mg EDTA/ml (SARSTEDT, Nümbrecht, Germany).

Whole blood was analyzed for hemoglobin, hematocrit, total counts of erythrocytes, red cell distribution width (RDW), mean corpuscular volume of erythrocytes (MCV), mean corpuscular hemoglobin of erythrocytes (MCH), mean corpuscular hemoglobin concentration of erythrocytes (MCHC); total counts of leucocytes; percentage of lymphocytes, percentage of eosinophilic granulocytes, percentage of basophilic granulocytes and atypical lymphocytes, total counts of thrombocytes, mean thrombocyte volume (MTV), thrombocrit and percentage of immature cells by an automatic hematology analyzer (ABX Pentra 60, Horiba ABX SAS, Montpellier, France).

### Isolation of leucocytes from intestinal epithelium and lamina propria

Tissue samples from mid jejunum (without PP) and ileum were dissected and stored on ice in sample bags which were filled with 1x PBS. For the isolation of intraepithelial cells (IELs) and cells from the lamina propria of jejunum and ileum, the Lamina Propria Dissociation Kit from Miltenyi Biotec (Bergisch Gladbach, Germany) was used. Briefly, one gram of the respective intestinal segment was cut into small pieces and transferred into a 50 ml tube. The sample was blended by 20 ml of a predigestion solution containing PBS-HEPES (1x PBS without Mg^2+^ and Ca^2+^ (Life Technologies™; Carlsbad, USA) containing 10 mM HEPES (Sigma Aldrich Chemie GmbH; Munich, Germany), 5 mM EDTA (life technologiesTM), 5% FBS (Merck KGaA; Darmstadt, Germany) and 1 mM DTT (Cell Signaling Technology; Danvers; USA). Samples were incubated in a water bath for 20 min at 37°C under gentle shaking. Thereafter, samples were vortexed and the predigestion solution was transferred into a new 50 ml tube which was equipped with MACS^®^ SmartStrainers (mash size 70 μm, Miltenyi Biotec) and stored on ice for the collection of IELs. Fresh predigestion solution (20 ml) was added to the tubes containing the tissue pieces. Samples were again incubated in a water bath for 20 min at 37°C with gentle shaking and then vortexed. The supernatant was collected once again for IEL purification. Tissue samples were blended by 20 ml PBS-HEPES incubated for 20 min in the 37°C warm water bath and vortexed. The supernatant was collected as before. Tissue samples were transferred in a gentle MACS C tube (Miltenyi Biotec) containing 2.35 ml of warmed Digestion solution (1x PBS with Ca^2+^ and Mg^2+^ and 10 mM HEPES, 5% FBS) and the enzymes D, R and A, which were provided by the kit. The samples were then incubated for 30 min at 37°C in a water bath and thereafter homogenized by a gentle MACS Dissociator (Miltenyi Biotec). Thereafter, the sample tubes were centrifuged at 300 × *g* for 1 min. Five ml PBS buffer consisting of 1x PBS without Ca^+^ and Mg^2+^ and 0.5% FBS were added. The cell suspension was separated from the tissue by MACS^®^ SmartStrainers and the tissue was rejected. The cell suspension was centrifuged at 300 × *g* for 10 min. The supernatant was discarded and the remaining pellet was resuspended in 5 ml antibody buffer (4.9 ml 1x PBS without Ca^2+^ and Mg^2+^, 10 mM HEPES, 0.1 ml FBS, 2mM EDTA).

The collected cell suspension gained from the different washing steps was used for the purification of the IEL. Initially, the cell suspension was stored 10 min on ice to support the sedimentation of tissue pieces and villi. Thereafter, the cell suspension was decanted in a new 50 ml tube. Samples were centrifuged for 10 min at 300 × *g* at room temperature. The supernatant was discarded and the pellet was blended with 5 ml antibody buffer.

### Isolation of intestinal leucocytes from jejunal Peyer’s patches

The isolation of cells from PP was conducted by a modified method of Liermann et al. (27). Depending on the size, three to six PP (14 ± 3 g) were cut into small pieces and transferred into a 50 ml tube. Tissue samples were blended with 35 ml PBS-DTT (PBS-HEPES, 2 mM DTT) and incubated for 20 min in a water bath (37°C). Samples were vortexed and after sedimentation of the tissue pieces the supernatant was discarded. The tissue samples were mixed with PBS-EDTA (PBS-HEPES, 1 mM EDTA). The samples were incubated in the water bath for 20 min. Thereafter, samples were vortexed and the supernatant was discarded. Forty milliliters RPMI medium were added and the tissue pieces were fragmented. The gained cell suspension was sieved by MACS SmartStrainers and transferred in a new 50-ml tube. Samples were centrifuged at 600 x *g* for 10 min at room temperature. The supernatant was discarded and the pellet was resuspended in 25 ml 30% Percoll (Merck KGaA). Samples were centrifuged for 15 min at 350 x *g* and room temperature. The supernatant was discarded and the pellet was blended with 3 ml antibody buffer.

### Isolation of cells from blood, thymus, spleen and mesenteric lymph nodes

Samples of thymus, spleen and MSL were dissected and stored on ice in 50-ml tubes containing cold PBS. Leucocytes from thymus, spleen and MSL were isolated according to a modified method of Liermann et al. (27). Briefly, tissue samples (thymus 12 ± 2 g; spleen 12 ± 3 g; MSL 9 ± 4 g) were dissected from surrounding fat and serosa and transferred in petri dishes containing cold 1x PBS (without Ca^2+^ and Mg^2+^). Tissue samples were fragmented by scalpel to release the cells. The cell suspension was sieved by MACS^®^ SmartStrainers and centrifuged at 300 × *g* for 10 min at room temperature. After discarding of the supernatant, the pellet was resuspended in 5 ml antibody buffer.

In case of the cell suspension of spleen, an erythrocyte lyses was conducted. The same procedure was used to isolate leucocytes from whole blood. The cell suspension of spleen or whole blood (5 ml) was mixed with 5 ml 1x PBS and centrifuged at 1000 × *g* for 10 min at 4°C. The supernatant was rejected and the pellet was resuspended in 5 ml distilled H_2_O. Five ml of 2x PBS (produced from 10x PBS diluted by distilled H_2_O) was added. Samples were gently shaken and centrifuged at 500 × *g* for 10 min at 4°C. The supernatant was discarded again, the pellet was resuspended with 3 ml distilled water and after shaking 3 ml 2x PBS was added. The sample was centrifuged at 300 × *g* for 10 min at 4°C. The supernatant was removed and the pellet was resuspended in 5 ml antibody buffer. If the cell pellet remained intensively red the last washing step was repeated once again before the pellet was diluted in antibody buffer.

### Flow cytometry

Antibody staining of cells and flow cytometric quantification of T and B cells were described in detail by Liermann et al. (28). Briefly, cells were stained by monoclonal antibodies for cluster of differentiation (CD)2 (T cells) (mouse anti-bovine CD2: FITC; Bio-Rad Laboratories, Hercules, USA), CD4 (T helper cells) (mouse anti-bovine CD4: FITC; Bio-Rad), CD8 (cytotoxic cells) (mouse anti-bovine CD8: Alexa Fluor^®^ 647; Bio-Rad), and CD21 (B cells) (mouse anti-bovine CD21: RPE, Bio-Rad) or the corresponding isotype controls (mouse IgG1 negative control: FITC; mouse IgG2a negative control: FITC; mouse IgG2a negative control: Alexa Fluor^®^ 647; mouse IgG2b negative control: RPE; Bio-Rad). A flow cytometer Type GalliosTM (Beckman Coulter GmbH, Krefeld Germany) was used.

### Quantitative real time PCR in the intestine and spleen

Tissue samples were collected from spleen as well as scraped jejunal mucosa which were closely located to the samples used for T and B cell quantification. Samples were immediately frozen in liquid nitrogen and stored at -80°C until further analyses. For the RNA extraction from spleen and jejunal mucosa, the innuPREP RNA Mini Kit 2.0 (Analytik Jena, Jena, Germany) was used and the relative gene expression was estimated by reverse-transcriptase quantitative PCR (RT-qPCR) as described by Koch et al. (29). The RIN factors for spleen and jejunal mucosa were on average 8.3 and 7.2, respectively. Data were quantified by qbase software (Biogazelle, Gent, Belgium) using *emerin* (*EMD*) and *tyrosine 3-monooxygenase/tryptophan 5-monooxygenase activation protein zeta* (*YWHAZ*) as reference genes for spleen (M-value 0.48; CV-value 0.17) as well as *EMD* and *peptidylprolyl isomerase A* (*PPIA*) as reference genes for jejunum (M-value 0.28; CV-value 0.10). Primer sequences of intestinal mucosa target genes are summarized in **Supplemental Table S3**, including genes involved in the barrier function of the intestine such as tight junction proteins, fatty acid binding proteins, different anti- and pro-inflammatory cytokines, toll like receptors (TLR), and other immune factors which are strongly related to the T and B cell function and response. Genes encoding the T and B cell function were also used to analyze the immune response in spleen. Gene expression data of jejunum and spleen were added as **Supplemental Data sheet 1** and **2**.

### Lactate and volatile fatty acid analyses in intestinal content

Intestinal content was collected from mid jejunum. The pH of the content was determined immediately after collection. Thereafter, the samples were frozen in aliquots at -20°C until further analyses.

The intestinal content was gently thawed, homogenized, and centrifuged at 3000 × *g* at room temperature for 5 to 10 min. The supernatant was divided in aliquots. Total lactate concentration was measured colorimetrically according to the method of Haacker et al. (30). Volatile fatty acids (VFA) including acetic, propionic, iso-butyric, butyric, iso-valeric, and valeric acid were determined by gas chromatography using a gas chromatograph (GC-2010 Plus, Shimadzu Corp., Kyoto, Japan) equipped with a flame ionization detector and a 25-m × 0.25-mm free fatty acid phase column (Roth, Karlsruhe, Germany) as described by Tümmler et al. (26).

### Plasma immunoglobulin A and secretory immunoglobulin A in spleen and intestinal content

One aliquot of the supernatant from the intestinal content was used for sIgA analyses. Spleen tissue frozen at -80°C was pulverized under liquid nitrogen, mixed with ice-cold PBS and homogenized by Precellys Evolution Homogeniser (Bertin Technologies, Montigny le Bretonneux, France). For the determination of IgA in plasma and sIgA in spleen and intestinal content commercial bovine specific ELISA kits were used (kit number for plasma: LS-F36793, LSBioscience, Seattle, USA; kit number for spleen and intestinal content: EKU07233, Biomatik, Kitchener, Canada). The analyses were conducted according to the manufacturer’s protocol using the following dilutions: 1:10.000 (plasma), 1:200 (spleen), 1:10 (intestinal content).

### Statistical analyses and calculations

SAS (SAS Institute Inc., Cary, USA, 152 Version 9.4) was used for statistical analyses. The data was analysed by a multifactorial variance analyses using the MIXED procedure. Fixed factors were “pre-weaning MR feeding intensity” (MRF, 20MR or 10MR) and “rumen cannula” (yes, RC or no, NRC) as well as their interactions. The potential impact of the dehorning process was additionally considered in the statistical model as a fixed effect (yes or no). Least squares means (LsMeans) and corresponding standard errors (SE) were calculated for each fixed factor. The Tuckey-Kramer procedure was conducted to test all pair-wise differences between LsMeans. Differences were declared as significant if *P* < 0.05 and a trend was defined by *P* < 0.1. If not stated otherwise, results are presented as LsMeans ± SE. Pearson correlation coefficients were calculated and declared significant at *P* < 0.05. The correlation power was estimated by the POWER procedure.

## Results

Heifers of the 20MR group were heavier (312 ± 7 kg) compared to heifers from the 10MR group (280 ± 7 kg) on slaughtering day (*P* < 0.05). There was no difference in the body weight between NRC and RC heifers. TMR intake in the last week before slaughter was 17.4 ± 0.7 kg fresh matter/day in the 20MR and 16.4 ± 0.8 kg fresh matter/day in the 10 MR group. No differences in TMR intake were determined between RC heifers (17.1 ± 0.8 kg fresh matter/day) and NRC heifers (16.7 ± 0.7 kg fresh matter/day).

### Hematology

Pooled LsMeans of MCV and MCH were higher in RC compared to NRC heifers (*P* < 0.01). A significant MRF by rumen cannulation interaction was detected in case of atypical lymphocytes showing the lowest number of these cell type in 10MRNRC heifers and the highest number in 10MRRC heifers.

Pooled LsMeans of MCHC were higher in 10MR than 20MR heifers (*P* = 0.022) (**Table 1**).

**Table 1:** Hematology of 34-week old heifers depending on pre-weaning feeding intensity and rumen cannulation (LsMeans ± SE).

Variable	Group				P- values		
	20MR^1^		10MR^2^				
	NRC^3^ (n = 5)	RC^4^ (n = 7)	NRC (n = 5)	RC (n = 7)	MRF^5^	RC	MRF x RC
Hematocrit, %	30.4 ± 1.3	30.0 ± 1.1	28.2 ± 1.3	31.1 ± 1.1	0.679	0.331	0.193
Hemoglobin, g/dl	9.5 ± 0.4	9.5 ± 0.3	9.2 ± 0.4	10.1 ± 0.3	0.685	0.309	0.249
Erythrocytes, 10^6^/mm^3^	7.7 ± 0.3	7.4 ± 0.3	7.7 ± 0.3	7.8 ± 0.3	0.575	0.735	0.380
RDW^6^, %	17.0 ± 0.5	17.6 ± 0.4	16.7 ± 0.5	17.1 ± 0.4	0.394	0.282	0.851
MCV^7^, µm^3^	39.2 ± 1.0	40.6 ± 0.9	36.7 ± 1.0	40.0 ± 0.9	0.137	0.033	0.352
MCH^8^, pg	12.2 ± 0.3	12.9 ± 0.3	12.1 ± 0.3	12.9 ± 0.3	0.876	0.019	0.705
MCHC^9^, g/dl	31.2 ± 0.5	31.7 ± 0.4	32.8 ± 0.5	32.4 ± 0.4	0.022	0.919	0.334
Leucocytes, 10^3^/mm^3^	12.6 ± 1.2	10.1 ± 1.0	9.9 ± 1.2	11.4 ± 1.0	0.512	0.687	0.079
Lymphocytes, %	34.0 ± 5.4	34.0 ± 4.6	36.3 ± 5.6	43.2 ± 4.5	0.265	0.509	0.492
Atypical Lymphocytes, %	2.4 ± 0.2^ab^	2.2 ± 0.2^ab^	2.0 ± 0.2^b^	2.7 ± 0.2^a^	0.808	0.186	0.015
Basophile Granulocytes, %	1.0 ± 0.2	1.2 ± 0.2	0.7 ± 0.2	1.0 ± 0.2	0.239	0.333	0.839
Thrombocytes, 10^3^/mm^3^	450 ± 68	421 ± 63	572 ± 78	440 ± 57	0.299	0.259	0.447
MTV^10^, µm^3^	7.0 ± 0.3	7.1 ± 0.3	7.8 ± 0.3	7.1 ± 0.3	0.209	0.299	0.234
Thrombocrit	0.32 ± 0.07	0.30 ± 0.06	0.46 ± 0.07	0.31 ± 0.06	0.241	0.231	0.303
Immature cells, %	2.4 ± 3.3	7.9 ± 2.7	2.6 ± 3.3	2.2 ± 2.7	0.367	0.418	0.333

^1^20MR, 20% per kg BW pre-weaning milk replacer feeding,

^2^10MR, 10% per kg BW pre-weaning milk replacer feeding.

^3^NRC, non-rumen cannula.

^4^RC, rumen cannula.

^5^MRF, milk replacer feeding.

^6^RDW, red cell distribution width.

^7^MCV, mean corpuscular volume of erythrocytes.

^8^MCH, mean corpuscular hemoglobin of erythrocytes.

^9^MCHC, mean corpuscular hemoglobin concentration of erythrocytes.

^10^MTV, mean thrombocyte volume.

^ab^Different superscripts mark significant differences between groups (P < 0.05).

### T and B cell subsets in MSL and intestinal epithelium, lamina propria and PP

**Figure 1:**
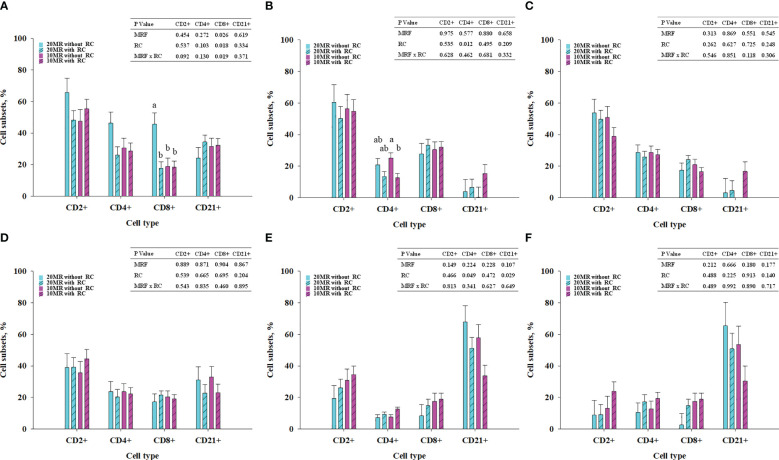
T and B cell subsets in mesenteric lymph nodes **(A)**, jejunal intraepithelial cells **(B)**, jejunal lamina propria **(C)**, jejunal Peyer´s patches **(D)**, ileal intraepithelial cells **(E)** and ileal lamina propria **(F)** depending on milk replacer feeding (blue bars, 20% milk replacer (MR); pink bars, 10% MR) and rumen cannulation (clear bars, no rumen cannula; striped bares, with rumen cannula) in 34-week old heifers (LSMeans ± SE). MRF, milk replacer feeding; RC, rumen cannula. ^ab^Different superscripts mark significant differences between groups at P < 0.05.

NRC Heifers fed 20MR showed higher CD8^+^ T cell subsets in MSL compared to all other heifer groups (*P* < 0.05) (**Figure 1A**). The CD8^+^ T cell subsets in MSL were positively correlated with CD2^+^ (*r* = 0.513; *P* = 0.021) and CD4^+^ T cell subsets (*r* = 0.878; *P* < 0.001) but negatively correlated with CD21^+^ B cell subsets (r = -0.691; P = 0.001).

Pooled LsMeans of the CD4^+^ T cell subsets in jejunal IELs were higher in NRC heifers compared to RC heifers (*P* = 0.012) (**Figure 1B**). A clear statistical significance was detected between NRC and RC heifers receiving 10% MR. The T and B cell subsets in the jejunal lamina propria and PP (**Figures 1C, D**) were not affected by rumen cannula or MRF. Pooled LsMeans of CD4^+^ T cell subsets in ileal IELs were lower in NRC compared to RC heifers (*P* < 0.05) (**Figure 1E**). Furthermore, rumen cannulation affected the CD21^+^ B cell subsets in ileal IELs (*P* = 0.029). Specifically, pooled LsMeans of CD21^+^ B cell subsets were higher in NRC compared to RC heifers. The T and B cell subsets in the ileal LP (**Figure 1F**) were not affected by fixed effects (*P* > 0.05).

### T and B cell subsets in blood, thymus and spleen

Blood T and B cell subsets were not affected by rumen cannula or MRF (*P* > 0.05) (**Table 2**). Subsets of CD21^+^ B cells in thymus tended to be higher in 20MRNRC heifers compared to 20MRRC heifers (*P* = 0.096). CD8^+^ T cell subsets tended to be lower in spleen of 20MRNRC heifers compared to all other groups (*P* < 0.1). Splenic CD21^+^ B cell subsets in 20MRNRC heifers were higher compared to RC heifers and tended to be higher compared to 10MRNRC heifers (*P* = 0.056). Splenic CD8^+^ T cell subsets were positively correlated with CD2^+^ T cell subsets (*r* = 0.544; *P* = 0.031) and CD4^+^ T cell subsets (*r* = 0.927; *P* < 0.001) but negatively correlated with CD21^+^ B cell subsets (*r* = -0.715; *P* < 0.001).

**Table 2:** T and B cell subsets in blood, thymus and spleen in 34-week old heifers depending on pre-weaning feeding intensity and rumen cannulation (LsMeans ± SE).

Variable	Group				P values		
	20MR^1^		10MR^2^				
	NRC^3^ (n = 5^6^)	RC^4^ (n = 7)	NRC (n = 5)	RC (n = 7)	MRF^5^	RC	MRF x RC
Blood							
CD2^+^ cells, %	8.1 ± 6.6	18.9 ± 5.1	13.0 ± 5.9	15.3 ± 4.8	0.909	0.301	0.445
CD4^+^ cells, %	34.4 ± 6.2	25.8 ± 4.7	18.4 ± 5.5	30.6 ± 4.4	0.291	0.747	0.059
CD8^+^ cells, %	6.6 ± 4.6	6.2 ± 2.9	10.5 ± 3.8	8.9 ± 2.9	0.381	0.790	0.883
CD21^+^ cells, %	44.9 ± 5.2	48.7 ± 4.0	49.3 ± 4.6	51.5 ± 3.7	0.420	0.533	0.857
Thymus							
CD2^+^ cells, %	75.1 ± 14.9	78.0 ± 11.3	61.9 ± 12.1	68.8 ± 9.7	0.363	0.712	0.867
CD4^+^ cells, %	59.4 ± 6.2	72.1 ± 4.7	65.9 ± 5.0	68.7 ± 4.0	0.758	0.166	0.323
CD8^+^ cells, %	54.4 ± 7.7	72.7 ± 4.3	69.1 ± 5.6	70.2 ± 4.1	0.301	0.112	0.141
CD21^+^ cells, %	11.8 ± 3.6	0.9 ± 2.4	3.6 ± 2.9	3.3 ± 2.3	0.322	0.077	0.073
Spleen							
CD2^+^ cells, %	56.5 ± 13.9	54.6 ± 9.3	66.1 ± 11.2	60.1 ± 9.0	0.496	0.736	0.851
CD4^+^ cells, %	46.5 ± 8.9	61.6 ± 6.8	64.9 ± 7.9	57.6 ± 5.8	0.342	0.629	0.144
CD8^+^ cells, %	32.5 ± 9.8	61.9 ± 5.4	66.3 ± 7.1	60.8 ± 5.3	0.038	0.122	0.026
CD21^+^ cells, %	27.9 ± 5.7^a^	7.6 ± 3.8^b^	7.9 ± 4.6^ab^	6.2 ± 3.7^b^	0.029	0.033	0.050

^1^20MR, 20% per kg BW pre-weaning milk replacer feeding,

^2^10MR, 10% per kg BW pre-weaning milk replacer feeding.

^3^NRC, non-rumen cannula.

^4^RC, rumen cannula.

^5^MRF, milk replacer feeding.

^6^Two animals had two be excluded from the analyses because of technical reasons.

^ab^ Different superscripts mark significant differences between groups at P < 0.05).

### Relative gene expression in jejunal mucosa

Pooled LsMeans of the *prostaglandin endoperoxide synthase 2* (*PTGES2*) gene expression encoding *cyclooxygenase 2* (*COX2*) were higher in RC heifers compared to NRC heifers (P < 0.05) (**Table 3**). Pooled LsMeans of the relative gene expression of *mucin 2* (*MUC2*) tended to be decreased in heifers of the 10MR group compared to 20MR heifers (*P* = 0.098).

**Table 3:** Relative gene expression of target genes encoding intestinal barrier function and pathways of T and B cell response in jejunal mucosa of 34-week old heifers depending on rumen cannulation and pre-weaning feeding intensity (LsMeans ± SE).

Gene name^6^	Group				P values		
	20MR^1^		10MR^2^				
	NRC^3^ (n = 5)	RC^4^ (n = 7)	NRC (n = 5)	RC (n = 7)	MRF^5^	RC	MRF x RC
*BOLA*	1.30 ± 0.46	2.28 ± 0.40	0.84 ± 0.48	1.39 ± 0.39	0.132	0.105	0.614
*BOLA-DRB3*	1.75 ± 0.90	2.71 ± 0.78	2.31 ± 0.93	3.07 ± 0.76	0.591	0.340	0.903
*CLDN1*	1.15 ± 0.12	0.93 ± 0.13	1.03 ± 0.14	1.11 ± 0.11	0.806	0.597	0.248
*CLDN4*	1.72 ± 0.57	2.01 ± 0.49	0.59 ± 0.59	1.83 ± 0.48	0.234	0.185	0.379
*FABP2*	1.20 ± 0.74	2.47 ± 0.64	0.93 ± 0.76	1.38 ± 0.62	0.338	0.249	0.548
*FABP6*	1.32 ± 0.47	1.85 ± 0.41	1.82 ± 0.49	1.22 ± 0.40	0.874	0.937	0.210
*FFAR1*	3.15 ± 1.39	3.56 ± 1.21	0.89 ± 1.44	3.48 ± 1.18	0.378	0.285	0.407
*FFAR2*	2.60 ± 1.07	2.90 ± 0.93	0.74 ± 1.11	2.86 ± 0.91	0.357	0.266	0.367
*FFAR3*	2.65 ± 1.34	3.36 ± 1.16	0.70 ± 1.39	3.51 ± 1.13	0.478	0.196	0.404
*FFAR4*	2.83 ± 1.21	3.26 ± 1.05	0.96 ± 1.26	3.12 ± 1.02	0.385	0.290	0.446
*FGL2*	1.41 ± 0.40	1.44 ± 0.35	0.68 ± 0.42	1.52 ± 0.34	0.394	0.280	0.285
*HCAR1*	3.19 ± 1.42	3.40 ± 1.23	0.81 ± 1.47	3.75 ± 1.20	0.454	0.270	0.310
*HCAR2*	2.70 ± 1.17	3.11 ± 1.01	0.86 ± 1.21	2.99 ± 0.99	0.383	0.283	0.432
*IDO1*	1.23 ± 1.17	1.11 ± 1.15	0.86 ± 1.17	1.02 ± 1.14	0.164	0.904	0.370
*IL1B*	1.05 ± 0.18	0.83 ± 0.16	1.15 ± 0.19	1.26 ± 0.15	0.128	0.753	0.343
*IL2*	1.10 ± 0.16	1.06 ± 0.14	0.94 ± 0.17	1.00 ± 0.14	0.465	0.972	0.735
*IL4*	1.32 ± 0.36	1.41 ± 0.32	0.74 ± 0.38	1.27 ± 0.31	0.303	0.393	0.530
*IL-6*	1.17 ± 0.23	0.95 ± 0.20	0.90 ± 0.24	1.35 ± 0.20	0.766	0.631	0.135
*IL10*	1.02 ± 0.16	1.04 ± 0.14	1.13 ± 0.17	1.02 ± 0.13	0.768	0.777	0.662
*IL17A*	2.58 ± 1.14	2.92 ± 0.99	0.81 ± 1.18	2.99 ± 0.96	0.435	0.274	0.394
*INFG*	1.21 ± 0.22	1.19 ± 0.19	0.97 ± 0.23	0.96 ± 0.19	0.271	0.930	0.989
*MUC2*	1.27 ± 0.25	1.32 ± 0.22	0.74 ± 0.26	1.04 ± 0.21	0.098	0.478	0.602
*NOS*	1.93 ± 0.60	1.69 ± 0.52	1.32 ± 0.63	0.96 ± 0.51	0.248	0.615	0.909
*OCLN*	0.87 ± 0.20	1.39 ± 0.18	1.00 ± 0.21	1.05 ± 0.17	0.575	0.171	0.222
*PTGES*	1.05 ± 0.11	0.92 ± 0.10	1.10 ± 0.12	1.05 ± 0.10	0.403	0.428	0.735
*PTGS1*	1.16 ± 0.24	1.36 ± 0.21	0.81 ± 0.25	1.09 ± 0.20	0.177	0.313	0.845
*PTGS2*	0.92 ± 0.16	1.31 ± 0.14	0.85 ± 0.16	1.12 ± 0.13	0.372	0.044	0.682
*RELA*	1.01 ± 0.06	0.96 ± 0.05	1.00 ± 0.06	1.02 ± 0.05	0.621	0.748	0.526
*SOD*	1.04 ± 0.12	1.11 ± 0.11	0.95 ± 0.13	1.03 ± 0.10	0.481	0.519	0.957
*TGFB1*	1.26 ± 0.21	0.87 ± 0.18	0.88 ± 0.22	1.20 ± 0.18	0.914	0.867	0.088
*TLR2*	2.19 ± 1.01	2.65 ± 0.88	0.82 ± 1.05	2.76 ± 0.85	0.512	0.241	0.437
*TLR3*	1.56 ± 0.62	1.91 ± 0.54	0.75 ± 0.64	1.79 ± 0.52	0.436	0.264	0.553
*TLR4*	1.32 ± 0.15	0.90 ± 0.13	1.00 ± 0.15	1.03 ± 0.12	0.517	0.195	0.106
*TLR6*	1.54 ± 0.54	1.71 ± 0.47	0.64 ± 0.56	1.73 ± 0.46	0.401	0.248	0.368
*TLR9*	3.12 ± 1.44	3.45 ± 1.25	0.76 ± 1.49	3.85 ± 1.21	0.477	0.241	0.309
*TLR10*	2.87 ± 1.33	3.31 ± 1.15	0.80 ± 1.38	3.40 ± 1.12	0.434	0.258	0.390
*TNFA*	1.09 ± 0.20	1.13 ± 0.17	0.82 ± 0.21	1.21 ± 0.17	0.605	0.295	0.345
*ZO1*	1.23 ± 0.36	1.42 ± 0.31	0.79 ± 0.37	1.32 ± 0.30	0.432	0.311	0.616
*ZO2*	1.00 ± 0.11	1.25 ± 0.10	0.98 ± 0.12	0.91 ± 0.09	0.102	0.414	0.130

^1^20MR, 20% per kg BW pre-weaning milk replacer feeding.

^2^10MR, 10% per kg BW pre-weaning milk replacer feeding.

^3^NRC, non-rumen cannula.

^4^RC, rumen cannula.

^5^MRF, milk replacer feeding.

^6^BOLA, major histocompatibility complex (MHC), class I; BOLA-DRB3, MHC class II; CLDN1, Claudin 1; CLDN4, Claudin 4; FABP2, fatty acid binding protein 2; FABP6, fatty acid binding protein 6; FFAR, free fatty acid receptor; FGL2, fibrinogen-like protein 2; MUC2, mucin2; NOS2, nitric oxide synthase 2; HCAR1, hydroxycarboxylic acid receptor 1; HCAR2, hydroxycarboxylic acid receptor 2; IDO1, indoleamine 2,3 diogygenase; IL, interleukin; INFG, interferon γ; OCLN, occluding; PTGES, prostaglandin endoperoxide Synthase; PTGES1; prostaglandin endoperoxide synthase 1 (Cox 1); PTGES2, Prostaglandin endoperoxide synthase 2 (Cox 2); RELA, Rela proto-oncogene/NF-κB subunit; SOD1, superoxide dismutase 1; TLR, toll-like receptor; TGFB1, transforming growth factor β 1; TNFA, tumor nekrosis factor α; ZO1, tight junction protein 1 (TJP1); ZO2, tight junction protein 2 (TJP2).

A significant correlation was found between the *PTGS2* expression and the *IL17A* expression in the jejunal mucosa (*r* = 0.719; *P* < 0.001).

### Relative gene expression in spleen

Pooled LsMeans of *TLR6* gene expression in RC heifers were higher compared to LsMeans in NRC heifers (*P* = 0.036) (**Table 4**). The pooled LsMeans of *IL4* also tended to be greater in RC heifers (*P* < 0.1).

**Table 4:** Relative gene expression of target genes encoding pathways of T and B cell response in spleen of 34-week old heifers depending on rumen cannulation and pre-weaning feeding intensity (LsMeans ± SE).

Gene name^6^	Group				P values		
	20MR^1^		10MR^2^				
	NRC^3^ (n = 5)	RC^4^ (n = 7)	NRC (n = 5)	RC (n = 7)	MRF^5^	RC	MRF x RC
*BOLA*	1.35 ± 0.47	2.41 ± 0.41	1.40 ± 0.49	1.19 ± 0.40	0.203	0.371	0.158
*BOlA-DRB3*	1.28 ± 0.88	3.14 ± 0.76	2.56 ± 0.91	2.44 ± 0.74	0.729	0.323	0.237
*FGL2*	0.94 ± 0.09	1.00 ± 0.08	1.23 ± 0.10	0.95 ± 0.08	0.191	0.233	0.061
*IDO1*	0.71 ± 0.45	1.04 ± 0.39	1.86 ± 0.47	1.06 ± 0.38	0.186	0.607	0.192
*IL1B*	0.88 ± 0.17	1.05 ± 0.15	1.05 ± 0.17	1.10 ± 0.14	0.495	0.498	0.705
*IL2*	0.81 ± 0.15	1.16 ± 0.13	1.01 ± 0.16	1.05 ± 0.13	0.754	0.199	0.276
*IL4*	0.81 ± 0.22	0.94 ± 0.19	0.70 ± 0.23	1.46 ± 0.19	0.341	0.058	0.153
*IL6*	0.93 ± 0.21	1.05 ± 0.18	1.25 ± 0.22	1.19 ± 0.18	0.267	0.893	0.630
*IL10*	0.82 ± 0.30	1.19 ± 0.26	1.23 ± 0.31	1.22 ± 0.25	0.443	0.537	0.496
*IL17A*	3.01 ± 1.22	2.84 ± 1.06	0.64 ± 1.27	1.29 ± 1.03	0.103	0.845	0.719
*INFG*	1.46 ± 0.35	1.08 ± 0.30	0.83 ± 0.36	1.43 ± 0.29	0.671	0.752	0.141
*PTGES*	0.90 ± 0.10	1.01 ± 0.09	1.15 ± 0.10	0.97 ± 0.08	0.272	0.683	0.129
*PTGES1*	1.06 ± 0.10	0.98 ± 0.08	0.96 ± 0.10	0.89 ± 0.08	0.296	0.453	0.920
*RELA*	1.01 ± 0.12	1.08 ± 0.10	1.09 ± 0.12	0.94 ± 0.10	0.818	0.722	0.314
*TGFB1*	0.87 ± 0.12	1.09 ± 0.10	1.07 ± 0.12	0.99 ± 0.10	0.667	0.547	0.182
*TLR2*	1.43 ± 0.30	1.63 ± 0.26	0.66 ± 0.31	0.99 ± 0.26	0.022	0.389	0.810
*TLR3*	1.49 ± 0.37	1.49 ± 0.31	0.74 ± 0.39	0.91 ± 0.31	0.072	0.818	0.802
*TLR4*	0.98 ± 0.13	1.06 ± 0.11	1.17 ± 0.14	0.95 ± 0.11	0.759	0.587	0.231
*TLR6*	0.81 ± 0.10	1.12 ± 0.12	0.97 ± 0.10	1.18 ± 0.12	0.340	0.036	0.665
*TLR9*	1.76 ± 0.48	1.36 ± 0.42	0.79 ± 0.50	1.15 ± 0.41	0.208	0.965	0.406
*TLR10*	1.69 ± 0.37	1.39 ± 0.32	0.75 ± 0.38	0.95 ± 0.31	0.058	0.897	0.474
*TNFA*	1.05 ± 0.16	1.11 ± 0.14	1.17 ± 0.16	0.97 ± 0.13	0.919	0.652	0.373

^1^20MR, 20% per kg BW pre-weaning milk replacer feeding.

^2^10MR, 10% per kg BW pre-weaning milk replacer feeding.

^3^NRC, non-rumen cannula.

^4^RC, rumen cannula.

^5^MRF, milk replacer feeding.

^6^BOLA, major histocompatibility complex (MHC), class I; BOLA-DRB3, MHC class II; FGL2, fibrinogen-like protein 2; IDO1, indoleamine 2,3 diogygenase; IL, interleukin; INFG, interferon γ; PTGES, prostaglandin endoperoxide synthase; PTGES1; prostaglandin endoperoxide synthase 1 (Cox 1); PTGES2, prostaglandin endoperoxide synthase 2 (Cox 2); RELA, Rela proto-oncogene/NF-κB subunit; SOD1, superoxide dismutase 1; TLR, toll-like receptor; TGFB1, transforming growth factor β 1; TNFA, tumor nekrosis factor α.

Pooled LsMeans of the relative gene expression of *TLR2* in 10MR heifers were reduced compared to 20MR heifers (*P* = 0.022). Furthermore, pooled LsMeans of the *TLR3* and *TLR10* gene expression tended to be lower in heifers with the low than with the high pre-weaning MR supply (*P* < 0.1).

There was a negative correlation between splenic CD8^+^ T cell subsets and *TLR2* (*r* = - 0.480; *P* = 0.032), and CD8^+^ T cell subsets tended to correlate negatively with *TLR10* (*r* = -0.421; *P* = 0.065). Furthermore, there was a positive correlation between splenic CD21^+^ B cell subsets and *TLR3* (r = 0.433; P = 0.044) and *TLR10* (*r* = 0.549; *P* = 0.008) and a trend for a positive correlation with *TLR2* (*r* = 0.409; *P* = 0.059).

### Physico-chemical characteristics of jejunal content

While pooled LsMeans of the pH in jejunal content were higher (*P* = 0.044), pooled LsMeans of dry matter content tended to be lower in NRC heifers compared to RC heifers (*P* = 0.091) (**Table 5**). Pooled LsMeans of acetic acid concentrations in intestinal content tended to be lower in 20MR heifers compared to those fed 10MR pre-weaning (*P* = 0.084).

**Table 5:** Physico-chemical characteristics of jejunal content depending on pre-weaning feeding intensity and rumen cannulation in 34-week old heifers (LsMeans ± SE).

Variable	Group				P values		
	20MR^1^		10MR^2^				
	NRC^3^ (n = 5)	RC^4^ (n = 7)	NRC (n = 5)	RC (n = 7)	MFR^5^	RC	MFR x RC
pH	7.3 ± 0.1	7.0 ± 0.1	7.3 ± 0.1	7.1 ± 0.1	0.308	0.044	0.751
Dry matter, %	6.3 ± 0.7	7.9 ± 0.6	6.8 ± 0.7	7.5 ± 0.6	0.982	0.091	0.418
Lactate, g/l	0.20 ± 0.03	0.22 ± 0.02	0.24 ± 0.03	0.22 ± 0.02	0.437	0.972	0.428
Acetic acid, mg/ml	0.53 ± 0.08	0.53 ± 0.07	0.69 ± 0.09	0.66 ± 0.07	0.084	0.847	0.874
Iso-butyric acid, mg/ml	0.09 ± 0.01	0.10 ± 0.01	0.11 ± 0.01	0.11 ± 0.01	0.143	0.822	0.624
Total volatile fatty acids, mg/ml	0.63 ± 0.11	0.62 ± 0.10	0.81 ± 0.12	0.80 ± 0.10	0.107	0.950	0.993

^1^20MR, 20% per kg BW pre-weaning milk replacer feeding.

^2^10MR, 10% per kg BW pre-weaning milk replacer feeding.

^3^NRC, non-rumen cannula.

^4^RC, rumen cannula.

^5^MRF, milk replacer feeding.

There was a significant correlation between the dry matter of intestinal content and the ileal CD4^+^ T cell subsets of the IELs (*r* = 0.530; *P* = 0.013) but not between the dry matter content and the jejunal CD4^+^ T cell subsets (*P* > 0.05).

### IgA concentrations in plasma, spleen and jejunal content

There were no effects of rumen cannulation or the pre-weaning feeding regime on total IgA concentrations in plasma or sIgA concentrations in spleen and jejunal content (**Table 6**) (*P* > 0.05). A positive correlation was detected between plasma IgA concentrations and jejunal sIgA concentrations (*r* = 0.452; *P* = 0.027).

**Table 6:** IgA concentration in plasma, spleen and intestinal content depending on the pre-weaning feeding intensity and rumen cannulation in 34-week old heifers (LsMeans ± SE).

Variable	Group				P- values		
	20MR^1^		10MR^2^				
	NRC^3^ (n = 5)	RC^4^ (n = 7)	NRC (n = 5)	RC (n = 7)	MRF^5^	RC	MRF x RC
Total IgA, ng/ml							
Plasma	381 ± 26	354 ± 22	350 ± 27	356 ± 22	0.569	0.675	0.490
Secretory IgA, ng/ml							
Spleen	1021 ± 145	1149 ± 126	961 ± 151	1275 ± 123	0.811	0.139	0.495
Jejunal content	4.89 ± 0.88	6.03 ± 0.76	5.64 ± 0.91	5.88 ± 0.74	0.723	0.436	0.590

^1^20MR, 20% per kg BW pre-weaning milk replacer feeding.

^2^10MR, 10% per kg BW pre-weaning milk replacer feeding.

^3^NRC, non-rumen cannula.

^4^RC, rumen cannula.

^5^MRF, milk replacer feeding.

## Discussion

The present study aimed to investigate effects of rumen cannulation in combination with two different pre-weaning milk feeding intensities on the intestinal immune system of heifers.

To our best knowledge this is the first study which revealed alterations in the T and B cell subsets of the local intestinal immune system in cattle equipped with a rumen cannula at an early age. Similarly, Subramaniam et al. (31) reported an impact of stomach manipulation in rats by sleeve gastrectomy or a gastric bypass on adaptive immune pathways in the jejunum, involving changes in *IL17A*, *IL23* and *interferon gamma* (*INFG)*, and *IL33*. IL17A is a pro-inflammatory cytokine which is released after Th17-helper cell stimulation by the cytokine IL-23 (32). In the present study, the gene expression of interleukins or *INFG* in jejunum was not affected by rumen cannulation; however, jejunal CD4^+^ T cell subsets which include also Th17-helper cells were reduced in RC heifers. Interestingly, in ileum an opposite effect on CD4^+^ T cells was observed. This opposite effect might be mainly based on the fact that the ileal tissue is lined by PP to a great extent and thus differs in its function and general immune cell distribution compared to the dissected jejunal tissue, which in turn was completely free of PP. This was also the reason why no separate PP analyses were performed for the ileum.

At the moment, it is not possible to define the direct causes of immune modulations in the intestinal epithelium by rumen cannulation. However, it is assumed that alterations in intestinal immune cell subsets are based on changes in physico-chemical characteristics of the intestinal content. This hypothesis is supported by the fact that immune modulations were only observed in the epithelium of the intestine, which is in direct contact with the intestinal content but not in the lamina propria or the PP. The hypothesis is further supported by the effects of rumen cannulation on the intestinal dry matter content and pH although a positive correlation was only detected between ileal CD4^+^ T cell subsets of the IELs and the dry matter content. In a pig study by Liermann et al. (27), relationships between dry matter content and pH of the stomach content and intestinal CD4^+^ and CD8^+^ T cell subsets were observed. Subramaniam et al. (31) mentioned bile acids, the microbiota and incretin hormones as potential factors affecting the cytokine profile. Indeed, Wang et al. (5) and Wang et al. (6) found an influence of rumen cannulation on ruminal microbiota in goats and the ruminal methanogen community in cows. These authors did not analyze the microbial community in the intestine but it is assumed that alterations in the upper gastrointestinal tract might also modulate the microbial community in the further aboral gastrointestinal tract. Interestingly, first results revealed differences concerning 61 genera of the adherent microbiota in jejunum of 10MRRC or 10MRNRC heifers, 13 genera in jejunum of 20MR heifers and 19 genera of the adherent microbiota in the ileum of heifers with and without rumen cannula in both feeding groups (W. Liermann and H. Reyer, FBN, unpublished data).

Furthermore, it was shown that stomach lesions in pigs are also associated with T cell modulations in the jejunum as well as MSL (27). Pigs with severe stomach lesions have increased CD4^+^ T cell subsets in the jejunal lamina propria compared to pigs with moderate stomach lesions (27). Furthermore, these authors showed lower CD4^+^ T cell subsets in pigs with stomach lesions compared to pigs without stomach lesions in jejunal PP (27). Perhaps there are some parallels between the stomach lesions and surgical rumen cannula implantation although rumen cannulation was conducted several month before sample collection.

The increase in *PTGS2* encoding the *COX2* enzyme might indicate a pathogenic process in the jejunum of RC heifers. *COX2* mainly acts in inflammatory processes associated with ischemia, wound, or ulcer healing (33). The *in vitro* and *in vivo* studies by Li et al. (**34**, **35**) demonstrated the regulatory effect of *COX2* on CD4^+^ cell differentiation. However, we observed no correlations between the jejunal *PTGS2* expression and the CD4^+^ T cell subsets in jejunum and ileum, but a significant correlation was found between the *PTGS2* and *IL17A* expression in the jejunal mucosa. As *COX2* seems to affect the Th17 T-cell differentiation of naive CD4^+^ cells (34), the role of Th17 T-helper cells, which are specific *IL17A* expressing T helper cells, should be considered in ongoing studies.

There was no direct effect of the MR feeding regime on local immune cell subsets in the intestine but in immune cell storage organs such as MSL CD8^+^ T cells were higher in 20MRNRC heifers. Perhaps the higher pre-weaning MFR might increase the defence potential of MSL enabling a fast and efficient response in case of a pathogenic situation. However, an increase of cytotoxic cells might also indicate a higher antigen exposure. Interestingly, the CD8^+^ T cell subsets in MSL of 10MRNRC heifers equalled CD8^+^ T cell subsets in RC animals.

The higher MRF seemed to affect *MUC2* gene expression in jejunum suggesting a higher mucin secretion in these animals. *MUC2* plays an important role in the epithelial barrier function and especially in the response to the microbiota (36). *Ad libitum* feeding of MR has been shown to increase villus circumferences and villus surface area in calf’s jejunum (16, 17). In neonatal piglets an increased nutrient intake increased the mucosal mass (37). Therefore, increased gene expression of *MUC2* after a higher pre-weaning MR supply in the present study might be also associated with an increase of mucosal mass of the jejunum. Indeed, a higher intestinal density, which is the mass-length ratio of the small intestine, was found in 20MR compared to 10MR heifers (W. Liermann, L. M. Tümmler and B. Kuhla, FBN, unpublished data). The effect of the pre-weaning MRF on the intestinal *MUC2* expression might be based on the sustaining trend of a higher TMR and solid feed intake in animals which received the higher MR supply during the pre-weaning feeding period as shown by Tümmler et al. (26), although in the days before slaughter no clear difference between feed intake of 20MR and 10MR heifers was observed. The differences in feed intake before and after the pre-weaning period as presented by Tümmler et al. (26) might also contribute to differences in the gastrointestinal content which is supported by the differences in the intestinal acetate concentration. The differences in the intestinal acetate concentrations might be also related to differences in ruminal acetate concentrations and rumen size development. Indeed, 10MR heifers showed a faster ruminal development although the greater ruminal size in these animals did not persist after weaning (26). In addition, 10MR heifers at 34 weeks of age had a lower acetate concentration in the jejunal content, but had a higher ruminal acetate concentration, at least at age of week 22 (26). However, there was no relationship between jejunal acetate concentrations and *MUC2* gene expression in jejunum. Furthermore, *MUC2* gene expression was not related to the T and B cell subsets in the intestine.

In contrast to the MSL, in spleen the CD8^+^ T cell subsets were reduced in 20MRNRC heifers compared to the other groups. Conspicuously, equally to 10MRRC and 20MRRC heifers, CD8^+^ T cells slightly increased and CD21^+^ B cells slightly decreased in the spleen of 10MRNRC heifers compared to 20MRNRC heifers. This means that a restrictive MFR during the pre-weaning period affected the T and B cell subsets in spleen and MSL in the same way as rumen cannulation. Similar effects were also shown in thymus although they were less pronounced than in the spleen. However, 10MR feeding in combination with rumen cannulation seemed to have no exacerbating influence on the CD8^+^ T cell subsets or CD21^+^ B cell subsets. According to the negative correlation between CD8^+^ and CD21^+^ cells in MSL and spleen, the increase or decrease of CD8^+^ cells occurred at the expense of the proportions of the CD21^+^ cells. The decrease of CD21^+^ B cells might also explain the decreased gene expression of *TLR 2*, *3* and *10* in spleen of 10MR heifers, which are, for example, expressed by B cells or dendritic cells (32). Additionally, positive correlations between CD21^+^ B cell subsets and the *TLR 2*, *3* and *10* were found. Furthermore, the greater portions of CD8^+^ T cells in spleen were related to lower *TLR 2* and *10* expressions. The higher *IL4* expression in RC heifers might be a counter regulatory mechanism to cope with the lower CD21^+^ B cells in spleen. IL-4 is known as an anti-inflammatory cytokine produced by T cells or mast cells and drive the production of T-Helper-2 cells and activation of B cells (32).

It was initially hypothesized that the local immune modulations provoked by rumen cannulation or MRF in early age might affect not only local tissues such as the intestine or the MSL but also induce systemic effects in heifers when they get older. Although, the tissue specific alterations in spleen might indicate a systemic effect of rumen cannulation and the feeding regime, the initial hypothesis cannot be completely confirmed, because alterations in T and B cell subsets were not observed in blood. In addition, no relations between the immune modulation triggered by the rumen cannula or MRF in spleen and the alterations in the intestine were found. Nevertheless, MCV and MCH levels were higher in RC animals compared to NRC animals. These results indicate that the average volume and the haemoglobin concentration of the erythrocytes were higher in RC heifers than in NRC heifers. The MCHC value was not affected by rumen cannulation; however the MCHC was higher in 10MR heifers compared to 20MR heifers meaning the average haemoglobin concentrations of erythrocytes were higher in 10MR heifers. The reason for the observed differences between the RC and NRC animals as well as between the feeding groups are still unclear.

The overall reason for the alterations in the tissue specific immune system of RC animals cannot be fully explored by the present study. However, as mentioned above it is possible that the differences in pre-weaning and after weaning feed intake as described by Tümmler et al. (26) might be one impact factor contributing to differences in physico-chemical conditions of gastrointestinal content or microbial community. It must be also emphasized that the rumen cannulation was conducted before the rumen has fully developed. An impact of the rumen cannula on the ruminal development was not observed in heifers investigated in the present study (26). However, Tümmler et al. (26) showed effects of the rumen cannula on the digestibility of the organic matter and the mean retention time of the gastrointestinal content. Additionally, the different medical treatments for example antibiotics during and after the surgery might have influenced the immune system in the short- and long-term as well as directly or indirectly by factors such as the microbiota.

## Conclusion

Rumen cannulation in an early stage of life sustainably modulates the T and B cell subsets in the down-streaming gastrointestinal tract of heifers and has also an influence on the T and B cell subsets in immune cell storage organs such as MSL, spleen and thymus. The present study reveals also effects of the pre-weaning milk feeding regime on the intestinal mucin secretion and the T and B cell subsets in MSL, spleen and thymus several month after weaning. Interestingly, in MSL, spleen, and thymus, the restrictive milk feeding regime evoked similar modulations of T and B cell subsets as the rumen cannulation in 10MR and 20MR did. The biological relevance and the direct causes of these alterations have to be studied in future experiments.

## Data availability statement

The original contributions presented in the study are included in the article/**Supplementary Materials**. Further inquiries can be directed to the corresponding author.

## Ethics statement

The animal study was reviewed and approved by Landesamt für Landwirtschaft, Lebensmittelsicherheit und Fischerei Mecklenburg-Vorpommern, Rostock (registration number 7221.3-1.1-009/16).

## Author contributions

Conceptualization: WL, BK, HH. Methodology: WL and TV. Investigation: WL, L-MT. Resources: BK, HH. Writing-Original Draft Preparation: WL. Writing-Review & Editing: BK, HH. Supervision: HH. Project Administration: BK, HH. Funding Acquisition: BK and HH. All authors contributed to the article and approved the submitted version.
