# Supplementary material for: Effects of rumen cannulation combined with different pre-weaning feeding intensities on the intestinal, splenic and thymic immune system in heifer calves several month after surgery
Source: Front Immunol. 2023 Apr 18;14:1160935. doi: 10.3389/fimmu.2023.1160935 (PMC10151785; doi:10.3389/fimmu.2023.1160935)
Supplement: Supplementary file 1 [file DataSheet_1.pdf]

Relative gene expression jejunum

Samples	MRF	Rumen cannula	Dehorning	BOLA CNRQ	BOLA_DRB CNRQ	CLDN1 CNRQ
50	10MR	no	yes	1.619E+000	2.391E-002	1.501E+000
51	20MR	yes	yes	3.833E+000	6.744E+000	1.134E+000
59	20MR	no	yes	1.974E+000	2.532E+000	7.982E-001
66	10MR	yes	yes	3.806E+000	1.989E+000	1.021E+000
69	10MR	no	yes	2.435E+000	2.018E+000	6.296E-001
163	20MR	no	no	1.881E-001	3.382E-002	1.757E+000
229	20MR	no	no	1.637E+000	3.730E+000	1.189E+000
265	20MR	yes	no	5.348E-001	3.943E-002	1.022E+000
268	10MR	yes	no	1.324E+000	4.570E+000	1.527E+000
734	10MR	yes	yes	2.825E+000	4.273E+000	1.447E+000
748	20MR	no	yes	1.847E+000	2.605E+000	1.287E+000
759	20MR	yes	no	1.431E+000	4.156E+000	9.640E-001
761	10MR	yes	no	2.171E-001	4.100E+000	1.128E+000
763	10MR	no	yes	4.531E-001	2.923E+000	7.773E-001
771	20MR	no	yes	1.627E+000	1.958E-001	6.216E-001
773	10MR	no	yes	1.841E+000	3.832E+000	8.470E-001
781	20MR	yes	yes	2.574E+000	2.645E+000	8.214E-001
858	20MR	yes	no	3.902E+000	3.489E-002	8.579E-001
893	10MR	yes	yes	4.852E-001	3.643E+000	9.013E-001
906	10MR	no	no	1.882E-001	3.828E+000	1.159E+000
911	10MR	yes	no	2.170E-001	2.517E+000	7.458E-001
924	20MR	yes	no	4.873E-001	2.019E-002	9.702E-001
949	10MR	yes	no	7.088E-002	4.147E-002	1.059E+000
953	20MR	yes	no	8.698E-001	4.276E+000	9.723E-001

CLDN4 CNRQ	COX1 CNRQ	COX2 CNRQ	EMD CNRQ	FABP2 CNRQ	FABP6 CNRQ	FFAR1 CNRQ
5.986E-001	1.088E+000	9.387E-001	8.257E-001	9.895E-001	1.346E-003	1.307E-001
2.732E-001	5.734E-001	6.184E-001	9.694E-001	3.330E-001	1.206E+000	6.975E-002
3.539E+000	1.687E+000	1.352E+000	1.105E+000	1.772E+000	2.561E-001	7.495E+000
1.004E+000	2.385E+000	6.645E-001	9.204E-001	4.843E+000	1.505E+000	1.409E-001
2.526E-001	8.958E-001	6.797E-001	9.755E-001	8.399E-001	2.205E+000	4.527E-001
1.436E-001	8.289E-001	7.112E-001	9.075E-001	5.898E-001	2.455E+000	9.936E-002
2.054E-001	6.121E-001	7.220E-001	9.515E-001	2.089E-001	1.531E+000	1.740E-001
2.974E+000	2.105E+000	1.651E+000	1.169E+000	2.284E+000	3.899E+000	6.607E+000
1.340E+000	6.009E-001	7.063E-001	1.013E+000	1.382E-001	4.446E-001	2.660E+000
1.147E+000	1.033E+000	1.143E+000	9.780E-001	1.530E+000	4.261E-001	1.238E+000
1.985E+000	1.183E+000	9.896E-001	9.926E-001	1.106E+000	9.588E-001	3.373E+000
1.334E+000	9.999E-001	1.232E+000	1.052E+000	6.800E-001	6.872E-001	2.598E+000
1.429E+000	8.830E-001	8.854E-001	1.175E+000	4.752E-001	1.167E+000	3.076E+000
2.574E+000	1.436E+000	1.050E+000	9.060E-001	1.112E+000	2.258E+000	5.204E+000
3.129E+000	1.735E+000	8.407E-001	1.003E+000	2.649E+000	1.214E+000	5.297E+000
3.989E-001	8.591E-001	5.691E-001	9.810E-001	1.812E+000	2.816E+000	7.481E-002
3.444E+000	1.381E+000	1.803E+000	1.042E+000	2.152E+000	1.383E+000	7.087E+000
3.109E+000	1.098E+000	1.571E+000	1.313E+000	2.731E+000	1.567E+000	6.308E+000
3.598E+000	1.250E+000	1.623E+000	1.087E+000	1.426E+000	1.941E+000	8.099E+000
3.547E-001	4.604E-001	1.031E+000	9.911E-001	9.527E-001	1.202E+000	5.933E-001
4.496E-001	3.447E-001	1.436E+000	8.997E-001	2.242E-001	2.786E+000	6.120E-001
2.720E-001	6.261E-001	1.249E+000	9.883E-001	8.502E-001	3.537E+000	1.720E-001
3.453E+000	9.186E-001	1.359E+000	9.463E-001	6.494E-001	4.417E-001	7.833E+000
1.471E+000	2.041E+000	1.029E+000	9.720E-001	7.217E+000	1.288E+000	8.316E-002

FFAR2 CNRQ	FFAR3 CNRQ	FFAR4 CNRQ	FGL2 CNRQ	HCAR1 CNRQ	HCAR2 CNRQ	IDO1 CNRQ
2.260E-001	1.257E-001	3.399E-001	6.598E-001	1.215E-001	1.596E-001	6.437E-001
1.315E-001	1.180E-001	1.543E-001	7.169E-001	8.147E-002	1.307E-001	1.018E+000
5.975E+000	5.974E+000	6.799E+000	2.638E+000	7.220E+000	6.308E+000	6.121E-001
1.766E-001	7.823E-002	2.134E-001	3.823E-001	1.290E-001	1.791E-001	5.962E-001
4.840E-001	2.083E-001	5.439E-001	4.119E-001	3.959E-001	5.936E-001	9.523E-001
1.451E-001	1.071E-001	1.304E-001	5.931E-001	9.821E-002	1.399E-001	7.916E-001
2.648E-001	2.510E-001	2.901E-001	6.479E-001	1.726E-001	1.988E-001	1.564E+000
5.388E+000	5.794E+000	6.075E+000	2.498E+000	6.190E+000	5.874E+000	1.017E+000
2.406E+000	2.484E+000	2.394E+000	1.528E+000	2.748E+000	2.349E+000	7.219E-001
1.255E+000	1.079E+000	1.280E+000	1.143E+000	1.313E+000	1.073E+000	1.555E+000
2.957E+000	2.932E+000	3.023E+000	1.276E+000	3.376E+000	2.889E+000	1.324E+000
2.358E+000	2.312E+000	2.665E+000	1.009E+000	2.523E+000	2.518E+000	8.340E-001
2.640E+000	2.610E+000	2.837E+000	1.287E+000	2.965E+000	2.659E+000	1.293E+000
3.759E+000	4.403E+000	4.421E+000	1.821E+000	4.869E+000	4.295E+000	7.235E-001
4.171E+000	4.673E+000	4.430E+000	2.101E+000	5.767E+000	4.493E+000	1.869E+000
1.142E-001	2.374E-001	1.496E-001	4.436E-001	7.349E-002	9.040E-002	8.806E-001
5.583E+000	6.637E+000	5.955E+000	2.357E+000	6.769E+000	6.051E+000	1.319E+000
5.075E+000	6.328E+000	6.011E+000	2.227E+000	5.888E+000	5.388E+000	8.730E-001
6.244E+000	9.488E+000	6.942E+000	2.868E+000	9.122E+000	6.616E+000	1.156E+000
6.057E-001	5.111E-001	8.481E-001	6.515E-001	6.845E-001	7.022E-001	1.156E+000
6.363E-001	6.654E-001	7.270E-001	5.198E-001	6.622E-001	8.192E-001	1.079E+000
2.106E-001	2.520E-001	2.443E-001	4.320E-001	1.865E-001	1.934E-001	1.748E+000
6.178E+000	7.469E+000	6.918E+000	2.708E+000	8.607E+000	6.742E+000	7.236E-001
4.848E-002	8.342E-002	1.678E-001	2.367E-001	6.956E-002	4.825E-002	8.694E-001

IL10 CNRQ	IL17A CNRQ	IL1b CNRQ	IL2 CNRQ	IL4 CNRQ	IL6 CNRQ	INFG CNRQ
7.759E-001	1.512E-001	1.902E+000	1.487E+000	7.531E-001	1.144E+000	4.655E-001
9.032E-001	1.176E-001	1.212E+000	2.081E+000	7.207E-001	1.963E+000	1.359E+000
8.409E-001	6.339E+000	8.077E-001	1.220E+000	1.140E+000	1.234E+000	6.595E-001
4.744E-001	1.923E-001	8.135E-001	1.039E+000	3.480E-001	5.200E-001	6.540E-001
1.516E+000	6.570E-001	5.574E-001	6.438E-001	6.846E-001	3.849E-001	1.391E+000
9.054E-001	1.424E-001	1.384E+000	1.448E+000	1.002E+000	1.510E+000	8.965E-001
1.201E+000	2.327E-001	1.101E+000	1.342E+000	1.018E+000	1.700E+000	1.628E+000
1.257E+000	5.524E+000	1.015E+000	8.334E-001	1.869E+000	9.512E-001	8.369E-001
1.481E+000	2.090E+000	1.279E+000	9.726E-001	6.207E-001	1.321E+000	5.447E-001
9.723E-001	1.040E+000	1.976E+000	1.425E+000	1.147E+000	1.300E+000	1.483E+000
1.170E+000	2.707E+000	1.077E+000	1.044E+000	2.267E+000	6.626E-001	1.570E+000
8.242E-001	2.529E+000	8.458E-001	7.196E-001	9.786E-001	7.490E-001	9.565E-001
1.176E+000	2.373E+000	1.735E+000	9.644E-001	5.982E-001	8.438E-001	1.250E+000
1.724E+000	3.930E+000	7.255E-001	8.768E-001	6.931E-001	3.857E-001	6.577E-001
1.010E+000	3.977E+000	7.761E-001	5.413E-001	1.082E+000	6.157E-001	1.262E+000
8.268E-001	5.249E-002	9.164E-001	9.399E-001	5.047E-001	9.401E-001	9.196E-001
1.470E+000	5.305E+000	5.393E-001	8.015E-001	5.452E-001	1.063E+000	1.265E+000
1.336E+000	5.164E+000	7.424E-001	9.461E-001	1.010E+000	1.099E+000	9.686E-001
1.193E+000	7.229E+000	8.894E-001	9.604E-001	2.839E+000	1.147E+000	7.768E-001
8.538E-001	7.495E-001	1.418E+000	9.765E-001	8.637E-001	1.231E+000	1.355E+000
5.211E-001	8.233E-001	1.159E+000	9.025E-001	1.562E+000	2.071E+000	1.288E+000
8.569E-001	2.682E-001	1.281E+000	9.320E-001	1.334E+000	7.687E-001	2.466E+000
1.335E+000	6.683E+000	1.046E+000	6.516E-001	1.814E+000	2.401E+000	7.171E-001
6.317E-001	8.167E-002	3.866E-001	8.853E-001	3.632E+000	4.840E-001	5.210E-001

LRP10 RG CNRQ	MUC CNRQ	NOS CNRQ	OCLN CNRQ	PTGES CNRQ	RELA CNRQ	SOD CNRQ
4.907E-001	6.028E-001	1.706E-001	1.112E+000	1.395E+000	1.071E+000	1.037E+000
4.978E-001	5.241E-001	1.477E+000	8.795E-001	1.411E+000	1.139E+000	6.852E-001
1.759E+000	1.848E+000	1.780E+000	6.587E-001	9.691E-001	1.005E+000	1.403E+000
6.690E-001	9.539E-001	1.076E+000	2.100E+000	9.218E-001	1.117E+000	1.337E+000
7.558E-001	6.532E-001	6.834E-001	1.555E+000	1.292E+000	8.988E-001	8.481E-001
5.847E-001	7.447E-001	1.441E+000	9.202E-001	9.320E-001	1.086E+000	8.172E-001
5.257E-001	9.182E-001	7.055E-001	7.374E-001	1.345E+000	1.102E+000	9.862E-001
2.097E+000	2.722E+000	2.538E+000	1.878E+000	9.735E-001	1.049E+000	1.107E+000
8.905E-001	6.089E-001	2.421E-001	3.930E-001	1.306E+000	1.157E+000	8.667E-001
7.616E-001	5.520E-001	2.871E-001	8.215E-001	1.232E+000	9.601E-001	1.133E+000
1.122E+000	1.185E+000	3.828E-001	9.569E-001	1.040E+000	9.574E-001	1.019E+000
1.016E+000	1.324E+000	3.462E-001	1.061E+000	9.552E-001	8.620E-001	7.923E-001
1.179E+000	1.392E+000	8.416E-001	7.702E-001	1.448E+000	1.213E+000	7.417E-001
1.497E+000	9.685E-001	2.059E+000	1.116E+000	1.106E+000	9.195E-001	9.648E-001
1.584E+000	1.638E+000	5.662E+000	1.148E+000	9.430E-001	8.807E-001	9.748E-001
6.558E-001	6.386E-001	4.377E+000	9.411E-001	7.412E-001	1.031E+000	7.673E-001
1.847E+000	9.932E-001	1.049E+000	8.530E-001	6.241E-001	8.237E-001	1.101E+000
1.926E+000	1.767E+000	1.858E+000	1.755E+000	7.772E-001	1.105E+000	1.275E+000
2.174E+000	1.918E+000	2.179E+000	1.266E+000	7.850E-001	1.052E+000	1.083E+000
6.379E-001	8.333E-001	2.847E-001	4.875E-001	9.574E-001	1.051E+000	1.157E+000
7.406E-001	1.232E+000	1.118E+000	1.321E+000	8.010E-001	8.124E-001	1.004E+000
7.907E-001	7.122E-001	2.107E+000	1.212E+000	9.378E-001	8.926E-001	9.160E-001
1.883E+000	6.274E-001	6.297E-001	5.785E-001	8.490E-001	8.209E-001	1.067E+000
9.260E-001	1.235E+000	1.510E+000	1.901E+000	7.899E-001	8.532E-001	1.873E+000

TGFB1 CNRQ	TLR10 CNRQ	TLR2 CNRQ	TLR3 CNRQ	TLR4 CNRQ	TLR6 CNRQ	TLR9 CNRQ	TNF CNRQ
6.295E-001	1.480E-001	2.059E-001	3.132E-001	1.128E+000	3.375E-001	1.347E-001	8.082E-001
1.090E+000	1.942E-001	1.591E-001	2.684E-001	1.228E+000	3.904E-001	1.218E-001	9.114E-001
1.276E+000	6.888E+000	5.151E+000	3.385E+000	1.478E+000	3.165E+000	7.310E+000	1.568E+000
9.126E-001	1.167E-001	1.799E-001	3.418E-001	7.088E-001	3.534E-001	9.963E-002	8.276E-001
6.348E-001	3.929E-001	5.361E-001	7.337E-001	7.140E-001	4.397E-001	2.228E-001	6.378E-001
7.394E-001	1.194E-001	1.634E-001	3.540E-001	8.995E-001	4.060E-001	1.004E-001	9.477E-001
2.378E+000	1.800E-001	2.075E-001	4.136E-001	1.376E+000	4.907E-001	1.607E-001	8.825E-001
9.850E-001	5.187E+000	4.905E+000	3.381E+000	1.056E+000	2.922E+000	6.122E+000	1.400E+000
1.484E+000	2.289E+000	2.245E+000	1.289E+000	9.999E-001	1.413E+000	3.083E+000	1.110E+000
8.547E-001	1.131E+000	1.108E+000	8.618E-001	1.078E+000	8.450E-001	1.471E+000	9.811E-001
8.618E-001	2.621E+000	2.204E+000	1.450E+000	1.208E+000	1.517E+000	3.518E+000	1.086E+000
1.306E+000	2.627E+000	2.188E+000	1.644E+000	7.957E-001	1.380E+000	2.288E+000	8.637E-001
2.687E+000	2.919E+000	2.129E+000	1.550E+000	1.168E+000	1.637E+000	2.894E+000	1.027E+000
7.730E-001	4.970E+000	3.698E+000	2.403E+000	9.995E-001	2.163E+000	5.447E+000	1.653E+000
8.485E-001	5.247E+000	3.635E+000	2.405E+000	1.596E+000	2.324E+000	5.369E+000	1.079E+000
7.575E-001	9.375E-002	1.310E-001	2.246E-001	5.266E-001	3.030E-001	1.376E-001	6.018E-001
8.324E-001	6.841E+000	4.828E+000	3.211E+000	9.707E-001	2.947E+000	7.322E+000	1.794E+000
9.146E-001	5.961E+000	4.900E+000	3.532E+000	7.584E-001	2.960E+000	5.468E+000	1.382E+000
9.256E-001	8.425E+000	6.468E+000	3.966E+000	9.321E-001	3.597E+000	9.816E+000	1.590E+000
1.060E+000	5.114E-001	7.474E-001	7.605E-001	1.553E+000	6.173E-001	3.908E-001	6.609E-001
8.021E-001	5.256E-001	7.644E-001	6.484E-001	6.267E-001	6.535E-001	5.235E-001	6.645E-001
8.143E-001	1.944E-001	2.652E-001	3.798E-001	5.668E-001	3.557E-001	1.705E-001	8.714E-001
9.327E-001	7.659E+000	6.011E+000	3.676E+000	1.744E+000	3.430E+000	8.235E+000	2.153E+000
7.154E-001	8.440E-002	9.496E-002	2.845E-001	9.595E-001	3.734E-001	1.012E-001	3.846E-001

YWHAZ CNRQ	ZO1 CNRQ	ZO2 CNRQ
1.211E+000	6.008E-001	1.117E+000
1.032E+000	4.250E-001	8.177E-001
9.050E-001	2.143E+000	8.727E-001
1.087E+000	8.354E-001	1.092E+000
1.025E+000	8.237E-001	1.002E+000
1.102E+000	4.657E-001	8.408E-001
1.051E+000	5.514E-001	8.544E-001
8.558E-001	2.600E+000	1.611E+000
9.869E-001	8.188E-001	4.556E-001
1.022E+000	6.498E-001	9.497E-001
1.007E+000	1.242E+000	1.120E+000
9.505E-001	1.218E+000	1.105E+000
8.512E-001	1.086E+000	8.162E-001
1.104E+000	1.694E+000	1.016E+000
9.968E-001	1.860E+000	1.362E+000
1.019E+000	5.011E-001	1.100E+000
9.600E-001	1.985E+000	1.073E+000
7.618E-001	2.245E+000	1.271E+000
9.198E-001	2.458E+000	1.352E+000
1.009E+000	7.093E-001	8.491E-001
1.112E+000	6.680E-001	9.300E-001
1.012E+000	4.465E-001	1.101E+000
1.057E+000	2.613E+000	7.065E-001
1.029E+000	6.564E-001	1.617E+000
