# Supplementary material for: Effects of rumen cannulation combined with different pre-weaning feeding intensities on the intestinal, splenic and thymic immune system in heifer calves several month after surgery
Source: Front Immunol. 2023 Apr 18;14:1160935. doi: 10.3389/fimmu.2023.1160935 (PMC10151785; doi:10.3389/fimmu.2023.1160935)
Supplement: Supplementary file 2 [file DataSheet_2.pdf]

Reletive gene expression spleen

Sample	MRF	Rumen ca	Dehorning	420 RF CNRQ	BOLA CNRQ	BOLA-DRB3 CNRQ	CLDN1 CNRQ
50	10MR	no	yes	1.500E+000	3.375E+000	2.771E-002	2.305E+000
51	20MR	yes	yes	1.355E+000	4.892E+000	5.999E+000	1.010E+000
59	20MR	no	yes	8.275E-001	1.920E+000	2.497E+000	1.363E+000
66	10MR	yes	yes	8.605E-001	1.529E+000	2.528E+000	9.393E-001
69	10MR	no	yes	8.124E-001	2.854E+000	3.531E+000	1.122E+000
163	20MR	no	no	1.080E+000	3.741E-001	2.018E-002	1.078E+000
229	20MR	no	no	9.313E-001	2.064E+000	2.155E+000	6.968E-001
265	20MR	yes	no	1.100E+000	6.855E-001	1.896E-002	6.307E-001
268	10MR	yes	no	8.664E-001	1.870E+000	2.126E+000	9.095E-001
734	10MR	yes	yes	9.775E-001	2.779E+000	3.160E+000	1.776E+000
748	20MR	no	yes	6.802E-001	2.097E+000	1.544E+000	5.297E-001
759	20MR	yes	no	1.017E+000	2.861E+000	6.393E+000	7.761E-001
761	10MR	yes	no	9.501E-001	3.914E-001	2.522E+000	9.762E-001
763	10MR	no	yes	7.871E-001	6.984E-001	3.974E+000	2.077E+000
771	20MR	no	yes	1.088E+000	9.326E-001	7.520E-001	2.655E+000
773	10MR	no	yes	6.767E-001	1.742E+000	4.380E+000	6.040E-001
781	20MR	yes	yes	7.604E-001	2.414E+000	2.219E+000	6.716E-001
858	20MR	yes	no	8.301E-001	2.508E+000	1.365E-002	1.001E+000
893	10MR	yes	yes	9.483E-001	6.818E-001	3.996E+000	8.022E-001
906	10MR	no	no	9.144E-001	3.158E-001	2.601E+000	9.564E-001
911	10MR	yes	no	8.100E-001	4.061E-001	2.129E+000	6.444E-001
924	20MR	yes	no	8.629E-001	5.822E-001	9.494E-003	4.495E-001
949	10MR	yes	no	9.572E-001	8.883E-004	4.851E-002	9.334E-001
953	20MR	yes	no	9.424E-001	9.225E-001	5.594E+000	8.489E-001

COX1 CNRQ	EIFK3bos CNRQ	EMD CNRQ	FGL2 CNRQ	IDO1 CNRQ	IL10 CNRQ	IL17A CNRQ
1.506E+000	1.665E+000	1.284E+000	1.599E+000	1.383E+000	1.294E+000	1.113E+000
1.300E+000	1.877E+000	8.612E-001	1.060E+000	1.126E+000	1.387E+000	1.151E+000
7.552E-001	1.088E+000	1.785E+000	7.030E-001	3.279E-001	4.031E-001	1.470E+000
1.210E+000	1.057E+000	9.854E-001	1.002E+000	5.477E-001	9.932E-001	3.033E+000
8.676E-001	1.224E+000	9.460E-001	1.307E+000	5.436E+000	1.606E+000	1.514E+000
1.306E+000	1.021E-001	1.135E+000	1.089E+000	7.506E-001	6.515E-001	4.349E-001
1.062E+000	9.841E-001	9.651E-001	9.348E-001	1.053E+000	1.711E+000	6.406E-001
8.627E-001	1.054E+000	9.600E-001	1.191E+000	8.053E-001	6.743E-001	1.908E-001
8.853E-001	8.345E-001	9.737E-001	1.008E+000	7.302E-001	1.231E+000	5.204E-001
8.498E-001	1.186E+000	1.049E+000	1.296E+000	1.110E+000	1.052E+000	6.728E-001
1.203E+000	1.181E+000	8.344E-001	8.586E-001	6.336E-001	2.850E-001	1.119E+001
9.693E-001	1.294E+000	1.040E+000	1.155E+000	1.006E+000	6.785E-001	1.291E-001
9.422E-001	9.585E-001	9.806E-001	7.604E-001	2.194E+000	1.815E+000	2.912E-001
7.751E-001	1.232E+000	1.136E+000	9.870E-001	8.124E-001	1.117E+000	1.606E+000
1.024E+000	1.163E+000	1.009E+000	1.159E+000	7.690E-001	9.791E-001	2.728E+000
8.390E-001	9.499E-001	9.440E-001	1.342E+000	6.135E-001	4.566E-001	2.127E+000
1.050E+000	1.139E+000	8.394E-001	7.412E-001	8.288E-001	1.269E+000	1.096E+001
9.216E-001	1.080E+000	8.578E-001	9.422E-001	9.003E-001	3.218E+000	2.599E+000
7.135E-001	9.658E-001	9.260E-001	8.499E-001	9.375E-001	1.708E+000	6.208E-001
9.023E-001	7.729E-001	9.181E-001	9.978E-001	9.670E-001	1.449E+000	1.122E+000
6.321E-001	6.288E-001	8.507E-001	7.123E-001	9.390E-001	5.672E-001	1.710E+000
8.550E-001	8.385E-001	9.217E-001	7.716E-001	8.421E-001	6.741E-001	1.681E-001
9.723E-001	1.083E+000	9.281E-001	9.943E-001	9.968E-001	1.257E+000	7.657E-001
7.925E-001	1.018E+000	8.607E-001	1.083E+000	1.881E+000	6.778E-001	3.852E-001

IL1beta CNRQ	IL4 CNRQ	INFG CNRQ	Il2 CNRQ	Il6 CNRQ	LRP 10 RF CNRQ
2.116E+000	6.142E-001	5.766E-001	1.846E+000	8.456E-001	1.314E+000
1.302E+000	1.091E+000	8.746E-001	1.907E+000	9.281E-001	8.632E-001
7.902E-001	4.769E-001	2.447E-001	5.724E-001	6.445E-001	1.677E+000
8.218E-001	1.782E+000	2.433E+000	7.913E-001	8.450E-001	1.206E+000
5.282E-001	7.052E-001	7.533E-001	1.019E+000	2.368E+000	1.041E+000
9.389E-001	1.110E+000	1.405E+000	1.002E+000	1.487E+000	1.039E+000
1.082E+000	9.178E-001	1.495E+000	8.499E-001	1.150E+000	1.061E+000
1.059E+000	1.016E+000	5.262E-001	1.210E+000	8.705E-001	9.197E-001
1.465E+000	3.909E-001	3.694E-001	7.517E-001	8.073E-001	8.913E-001
1.419E+000	9.990E-001	7.490E-001	1.254E+000	1.058E+000	1.022E+000
3.759E-001	7.367E-001	2.833E+000	5.473E-001	4.544E-001	1.593E+000
1.297E+000	1.051E+000	5.445E-001	1.085E+000	1.290E+000	1.060E+000
1.228E+000	1.348E+000	1.073E+000	1.218E+000	1.257E+000	9.164E-001
9.793E-001	1.955E-001	2.697E-001	1.001E+000	5.343E-001	1.191E+000
1.182E+000	7.354E-001	1.327E+000	1.164E+000	8.126E-001	1.260E+000
6.218E-001	7.891E-001	5.332E-001	6.562E-001	9.514E-001	9.544E-001
9.916E-001	8.106E-001	1.494E+000	1.044E+000	1.471E+000	1.717E+000
1.033E+000	4.659E-001	5.948E-001	9.696E-001	8.783E-001	1.098E+000
9.422E-001	1.918E+000	2.309E+000	1.432E+000	1.339E+000	1.017E+000
9.471E-001	1.053E+000	2.078E+000	8.473E-001	1.260E+000	8.788E-001
8.029E-001	9.679E-001	1.989E+000	7.648E-001	1.306E+000	8.662E-001
7.813E-001	1.137E+000	1.851E+000	7.723E-001	4.987E-001	8.294E-001
1.031E+000	2.839E+000	1.029E+000	1.025E+000	1.786E+000	9.355E-001
9.200E-001	1.178E+000	1.572E+000	8.178E-001	1.745E+000	1.075E+000

PPIA CNRQ	PTGES CNRQ	RELA CNRQ	TGFB1 CNRQ	TLR10 CNRQ	TLR2 CNRQ	TLR3 CNRQ
7.786E-001	1.326E+000	1.766E+000	1.473E+000	1.002E+000	9.779E-001	8.147E-001
1.161E+000	1.166E+000	1.545E+000	1.776E+000	1.275E+000	2.726E+000	9.456E-001
5.603E-001	1.170E+000	1.126E+000	8.583E-001	9.182E-001	8.830E-001	8.067E-001
1.015E+000	8.412E-001	9.567E-001	9.918E-001	2.039E+000	1.558E+000	1.256E+000
1.057E+000	1.587E+000	1.090E+000	1.508E+000	8.968E-001	9.363E-001	9.614E-001
8.809E-001	9.019E-001	1.156E+000	8.340E-001	9.967E-001	8.024E-001	9.501E-001
1.036E+000	7.303E-001	1.066E+000	9.510E-001	9.248E-001	9.099E-001	7.617E-001
1.042E+000	8.761E-001	1.060E+000	8.824E-001	9.744E-001	7.616E-001	7.785E-001
1.027E+000	6.351E-001	8.986E-001	7.746E-001	6.797E-001	6.574E-001	5.850E-001
9.535E-001	1.190E+000	1.114E+000	1.103E+000	7.108E-001	7.566E-001	8.368E-001
1.198E+000	6.121E-001	6.197E-001	6.256E-001	4.527E+000	3.715E+000	3.898E+000
9.617E-001	1.170E+000	1.157E+000	1.153E+000	8.564E-001	7.328E-001	6.798E-001
1.020E+000	1.109E+000	1.107E+000	9.822E-001	4.959E-001	7.777E-001	4.481E-001
8.804E-001	1.121E+000	1.213E+000	1.096E+000	8.256E-001	8.004E-001	1.041E+000
9.914E-001	1.167E+000	1.122E+000	1.204E+000	1.470E+000	1.328E+000	1.424E+000
1.059E+000	7.975E-001	7.961E-001	8.201E-001	1.159E+000	1.029E+000	1.129E+000
1.191E+000	9.600E-001	9.214E-001	7.585E-001	3.138E+000	3.090E+000	3.981E+000
1.166E+000	9.697E-001	9.671E-001	9.567E-001	9.975E-001	1.210E+000	1.288E+000
1.080E+000	1.025E+000	8.285E-001	1.098E+000	6.364E-001	9.459E-001	7.265E-001
1.089E+000	1.111E+000	7.782E-001	7.712E-001	1.090E+000	9.402E-001	9.163E-001
1.176E+000	7.623E-001	7.171E-001	7.570E-001	8.416E-001	9.144E-001	1.155E+000
1.085E+000	8.240E-001	7.479E-001	8.543E-001	5.272E-001	7.920E-001	6.289E-001
1.077E+000	1.158E+000	8.864E-001	1.137E+000	8.248E-001	8.247E-001	9.718E-001
1.162E+000	9.138E-001	9.405E-001	9.671E-001	7.472E-001	6.808E-001	9.366E-001

TLR4 CNRQ	TLR6 CNRQ	TLR9 CNRQ	TNFa CNRQ
1.802E+000	1.197E+000	1.052E+000	2.060E+000
1.196E+000	1.070E+000	9.298E-001	1.632E+000
8.357E-001	6.781E-001	7.694E-001	9.710E-001
8.409E-001	1.083E+000	3.211E+000	1.086E+000
1.237E+000	1.225E+000	1.097E+000	1.259E+000
1.310E+000	7.613E-001	6.202E-001	1.198E+000
1.071E+000	4.004E-001	8.149E-001	1.443E+000
1.157E+000	7.679E-001	5.984E-001	1.102E+000
8.387E-001	8.310E-001	5.931E-001	8.936E-001
1.150E+000	1.337E+000	5.433E-001	1.336E+000
6.186E-001	1.461E+000	5.391E+000	5.324E-001
1.433E+000	1.197E+000	7.513E-001	1.124E+000
9.090E-001	6.829E-001	8.434E-001	1.028E+000
9.995E-001	8.168E-001	7.173E-001	1.207E+000
1.089E+000	8.846E-001	1.756E+000	1.191E+000
9.018E-001	1.185E+000	1.434E+000	8.149E-001
9.531E-001	1.527E+000	3.377E+000	9.977E-001
6.049E-001	8.334E-001	8.848E-001	1.011E+000
1.029E+000	1.319E+000	8.917E-001	7.427E-001
9.231E-001	8.349E-001	1.303E+000	7.231E-001
8.499E-001	1.445E+000	7.019E-001	8.144E-001
6.659E-001	8.769E-001	6.309E-001	5.763E-001
1.039E+000	1.442E+000	7.292E-001	7.954E-001
1.414E+000	1.156E+000	7.281E-001	1.141E+000
