# Supplementary material for: Effects of rumen cannulation combined with different pre-weaning feeding intensities on the intestinal, splenic and thymic immune system in heifer calves several month after surgery
Source: Front Immunol. 2023 Apr 18;14:1160935. doi: 10.3389/fimmu.2023.1160935 (PMC10151785; doi:10.3389/fimmu.2023.1160935)
Supplement: Supplementary file 3 [file DataSheet_3.pdf]

Samples	420 RF CNRQ	BOLA CNRQ	BOLA-DRB3 CNRQ	CLDN1 CNRQ	COX1 CNRQ	EIFK3bos CNRQ
163	1.080E+000	3.741E-001	2.018E-002	1.078E+000	1.306E+000	1.021E-001
229	9.313E-001	2.064E+000	2.155E+000	6.968E-001	1.062E+000	9.841E-001
265	1.100E+000	6.855E-001	1.896E-002	6.307E-001	8.627E-001	1.054E+000
268	8.664E-001	1.870E+000	2.126E+000	9.095E-001	8.853E-001	8.345E-001
50	1.500E+000	3.375E+000	2.771E-002	2.305E+000	1.506E+000	1.665E+000
51	1.355E+000	4.892E+000	5.999E+000	1.010E+000	1.300E+000	1.877E+000
59	8.275E-001	1.920E+000	2.497E+000	1.363E+000	7.552E-001	1.088E+000
66	8.605E-001	1.529E+000	2.528E+000	9.393E-001	1.210E+000	1.057E+000
69	8.124E-001	2.854E+000	3.531E+000	1.122E+000	8.676E-001	1.224E+000
734	9.775E-001	2.779E+000	3.160E+000	1.776E+000	8.498E-001	1.186E+000
748	6.802E-001	2.097E+000	1.544E+000	5.297E-001	1.203E+000	1.181E+000
759	1.017E+000	2.861E+000	6.393E+000	7.761E-001	9.693E-001	1.294E+000
761	9.501E-001	3.914E-001	2.522E+000	9.762E-001	9.422E-001	9.585E-001
763	7.871E-001	6.984E-001	3.974E+000	2.077E+000	7.751E-001	1.232E+000
771	1.088E+000	9.326E-001	7.520E-001	2.655E+000	1.024E+000	1.163E+000
773	6.767E-001	1.742E+000	4.380E+000	6.040E-001	8.390E-001	9.499E-001
781	7.604E-001	2.414E+000	2.219E+000	6.716E-001	1.050E+000	1.139E+000
858	8.301E-001	2.508E+000	1.365E-002	1.001E+000	9.216E-001	1.080E+000
893	9.483E-001	6.818E-001	3.996E+000	8.022E-001	7.135E-001	9.658E-001
906	9.144E-001	3.158E-001	2.601E+000	9.564E-001	9.023E-001	7.729E-001
911	8.100E-001	4.061E-001	2.129E+000	6.444E-001	6.321E-001	6.288E-001
924	8.629E-001	5.822E-001	9.494E-003	4.495E-001	8.550E-001	8.385E-001
949	9.572E-001	8.883E-004	4.851E-002	9.334E-001	9.723E-001	1.083E+000
953	9.424E-001	9.225E-001	5.594E+000	8.489E-001	7.925E-001	1.018E+000
IRC I	9.943E-001	9.290E-001	4.823E+000	9.832E-001	1.205E+000	1.364E+000
IRC II	1.140E+000	2.080E+000	5.051E+000	1.544E+000	1.178E+000	1.249E+000
IRC III	6.086E+000	2.022E+000	3.551E+000	1.627E+000	2.655E+000	1.197E+000
NTC	NaN	NaN	NaN	NaN	NaN	NaN

EMD CNRQ	FGL2 CNRQ	IDO1 CNRQ	IL10 CNRQ	IL17A CNRQ	IL1beta CNRQ	IL4 CNRQ
1.135E+000	1.089E+000	7.506E-001	6.515E-001	4.349E-001	9.389E-001	1.110E+000
9.651E-001	9.348E-001	1.053E+000	1.711E+000	6.406E-001	1.082E+000	9.178E-001
9.600E-001	1.191E+000	8.053E-001	6.743E-001	1.908E-001	1.059E+000	1.016E+000
9.737E-001	1.008E+000	7.302E-001	1.231E+000	5.204E-001	1.465E+000	3.909E-001
1.284E+000	1.599E+000	1.383E+000	1.294E+000	1.113E+000	2.116E+000	6.142E-001
8.612E-001	1.060E+000	1.126E+000	1.387E+000	1.151E+000	1.302E+000	1.091E+000
1.785E+000	7.030E-001	3.279E-001	4.031E-001	1.470E+000	7.902E-001	4.769E-001
9.854E-001	1.002E+000	5.477E-001	9.932E-001	3.033E+000	8.218E-001	1.782E+000
9.460E-001	1.307E+000	5.436E+000	1.606E+000	1.514E+000	5.282E-001	7.052E-001
1.049E+000	1.296E+000	1.110E+000	1.052E+000	6.728E-001	1.419E+000	9.990E-001
8.344E-001	8.586E-001	6.336E-001	2.850E-001	1.119E+001	3.759E-001	7.367E-001
1.040E+000	1.155E+000	1.006E+000	6.785E-001	1.291E-001	1.297E+000	1.051E+000
9.806E-001	7.604E-001	2.194E+000	1.815E+000	2.912E-001	1.228E+000	1.348E+000
1.136E+000	9.870E-001	8.124E-001	1.117E+000	1.606E+000	9.793E-001	1.955E-001
1.009E+000	1.159E+000	7.690E-001	9.791E-001	2.728E+000	1.182E+000	7.354E-001
9.440E-001	1.342E+000	6.135E-001	4.566E-001	2.127E+000	6.218E-001	7.891E-001
8.394E-001	7.412E-001	8.288E-001	1.269E+000	1.096E+001	9.916E-001	8.106E-001
8.578E-001	9.422E-001	9.003E-001	3.218E+000	2.599E+000	1.033E+000	4.659E-001
9.260E-001	8.499E-001	9.375E-001	1.708E+000	6.208E-001	9.422E-001	1.918E+000
9.181E-001	9.978E-001	9.670E-001	1.449E+000	1.122E+000	9.471E-001	1.053E+000
8.507E-001	7.123E-001	9.390E-001	5.672E-001	1.710E+000	8.029E-001	9.679E-001
9.217E-001	7.716E-001	8.421E-001	6.741E-001	1.681E-001	7.813E-001	1.137E+000
9.281E-001	9.943E-001	9.968E-001	1.257E+000	7.657E-001	1.031E+000	2.839E+000
8.607E-001	1.083E+000	1.881E+000	6.778E-001	3.852E-001	9.200E-001	1.178E+000
1.149E+000	1.536E+000	1.013E+000	1.203E+000	9.411E-001	1.047E+000	1.828E+000
1.313E+000	1.194E+000	9.671E-001	1.178E+000	9.844E-001	1.348E+000	8.761E-001
9.506E-001	5.439E-001	2.420E+000	1.088E+000	1.015E+000	1.431E+000	1.157E+001
NaN	NaN	NaN	NaN	NaN	NaN	NaN

INFG CNRQ	Interleukin2 CNRQ	Interleukin6 CNRQ	LRP 10 RF CNRQ	PPIA CNRQ	PTGES CNRQ
1.405E+000	1.002E+000	1.487E+000	1.039E+000	8.809E-001	9.019E-001
1.495E+000	8.499E-001	1.150E+000	1.061E+000	1.036E+000	7.303E-001
5.262E-001	1.210E+000	8.705E-001	9.197E-001	1.042E+000	8.761E-001
3.694E-001	7.517E-001	8.073E-001	8.913E-001	1.027E+000	6.351E-001
5.766E-001	1.846E+000	8.456E-001	1.314E+000	7.786E-001	1.326E+000
8.746E-001	1.907E+000	9.281E-001	8.632E-001	1.161E+000	1.166E+000
2.447E-001	5.724E-001	6.445E-001	1.677E+000	5.603E-001	1.170E+000
2.433E+000	7.913E-001	8.450E-001	1.206E+000	1.015E+000	8.412E-001
7.533E-001	1.019E+000	2.368E+000	1.041E+000	1.057E+000	1.587E+000
7.490E-001	1.254E+000	1.058E+000	1.022E+000	9.535E-001	1.190E+000
2.833E+000	5.473E-001	4.544E-001	1.593E+000	1.198E+000	6.121E-001
5.445E-001	1.085E+000	1.290E+000	1.060E+000	9.617E-001	1.170E+000
1.073E+000	1.218E+000	1.257E+000	9.164E-001	1.020E+000	1.109E+000
2.697E-001	1.001E+000	5.343E-001	1.191E+000	8.804E-001	1.121E+000
1.327E+000	1.164E+000	8.126E-001	1.260E+000	9.914E-001	1.167E+000
5.332E-001	6.562E-001	9.514E-001	9.544E-001	1.059E+000	7.975E-001
1.494E+000	1.044E+000	1.471E+000	1.717E+000	1.191E+000	9.600E-001
5.948E-001	9.696E-001	8.783E-001	1.098E+000	1.166E+000	9.697E-001
2.309E+000	1.432E+000	1.339E+000	1.017E+000	1.080E+000	1.025E+000
2.078E+000	8.473E-001	1.260E+000	8.788E-001	1.089E+000	1.111E+000
1.989E+000	7.648E-001	1.306E+000	8.662E-001	1.176E+000	7.623E-001
1.851E+000	7.723E-001	4.987E-001	8.294E-001	1.085E+000	8.240E-001
1.029E+000	1.025E+000	1.786E+000	9.355E-001	1.077E+000	1.158E+000
1.572E+000	8.178E-001	1.745E+000	1.075E+000	1.162E+000	9.138E-001
6.477E-001	1.025E+000	1.125E+000	4.443E-002	8.702E-001	1.073E+000
4.469E-001	1.167E+000	1.444E+000	3.059E+000	7.617E-001	1.417E+000
6.225E+000	1.574E+000	3.554E-001	1.236E+000	1.052E+000	1.068E+000
NaN	NaN	NaN	NaN	NaN	NaN

RELA CNRQ	TGFB1 CNRQ	TLR10 CNRQ	TLR2 CNRQ	TLR3 CNRQ	TLR4 CNRQ	TLR6 CNRQ	TLR9 CNRQ
1.156E+000	8.340E-001	9.967E-001	8.024E-001	9.501E-001	1.310E+000	7.613E-001	6.202E-001
1.066E+000	9.510E-001	9.248E-001	9.099E-001	7.617E-001	1.071E+000	4.004E-001	8.149E-001
1.060E+000	8.824E-001	9.744E-001	7.616E-001	7.785E-001	1.157E+000	7.679E-001	5.984E-001
8.986E-001	7.746E-001	6.797E-001	6.574E-001	5.850E-001	8.387E-001	8.310E-001	5.931E-001
1.766E+000	1.473E+000	1.002E+000	9.779E-001	8.147E-001	1.802E+000	1.197E+000	1.052E+000
1.545E+000	1.776E+000	1.275E+000	2.726E+000	9.456E-001	1.196E+000	1.070E+000	9.298E-001
1.126E+000	8.583E-001	9.182E-001	8.830E-001	8.067E-001	8.357E-001	6.781E-001	7.694E-001
9.567E-001	9.918E-001	2.039E+000	1.558E+000	1.256E+000	8.409E-001	1.083E+000	3.211E+000
1.090E+000	1.508E+000	8.968E-001	9.363E-001	9.614E-001	1.237E+000	1.225E+000	1.097E+000
1.114E+000	1.103E+000	7.108E-001	7.566E-001	8.368E-001	1.150E+000	1.337E+000	5.433E-001
6.197E-001	6.256E-001	4.527E+000	3.715E+000	3.898E+000	6.186E-001	1.461E+000	5.391E+000
1.157E+000	1.153E+000	8.564E-001	7.328E-001	6.798E-001	1.433E+000	1.197E+000	7.513E-001
1.107E+000	9.822E-001	4.959E-001	7.777E-001	4.481E-001	9.090E-001	6.829E-001	8.434E-001
1.213E+000	1.096E+000	8.256E-001	8.004E-001	1.041E+000	9.995E-001	8.168E-001	7.173E-001
1.122E+000	1.204E+000	1.470E+000	1.328E+000	1.424E+000	1.089E+000	8.846E-001	1.756E+000
7.961E-001	8.201E-001	1.159E+000	1.029E+000	1.129E+000	9.018E-001	1.185E+000	1.434E+000
9.214E-001	7.585E-001	3.138E+000	3.090E+000	3.981E+000	9.531E-001	1.527E+000	3.377E+000
9.671E-001	9.567E-001	9.975E-001	1.210E+000	1.288E+000	6.049E-001	8.334E-001	8.848E-001
8.285E-001	1.098E+000	6.364E-001	9.459E-001	7.265E-001	1.029E+000	1.319E+000	8.917E-001
7.782E-001	7.712E-001	1.090E+000	9.402E-001	9.163E-001	9.231E-001	8.349E-001	1.303E+000
7.171E-001	7.570E-001	8.416E-001	9.144E-001	1.155E+000	8.499E-001	1.445E+000	7.019E-001
7.479E-001	8.543E-001	5.272E-001	7.920E-001	6.289E-001	6.659E-001	8.769E-001	6.309E-001
8.864E-001	1.137E+000	8.248E-001	8.247E-001	9.718E-001	1.039E+000	1.442E+000	7.292E-001
9.405E-001	9.671E-001	7.472E-001	6.808E-001	9.366E-001	1.414E+000	1.156E+000	7.281E-001
1.153E+000	1.392E+000	1.317E+000	8.649E-001	1.066E+000	1.310E+000	1.302E+000	1.046E+000
1.368E+000	1.730E+000	1.155E+000	9.416E-001	1.024E+000	1.447E+000	1.328E+000	1.176E+000
6.850E-001	6.178E-001	4.398E-001	4.134E-001	9.868E-001	4.998E-001	6.378E-001	6.244E-001
NaN	NaN	NaN	NaN	NaN	NaN	NaN	NaN

TNF Nr.296 CNRQ

1.198E+000
1.443E+000
1.102E+000
8.936E-001
2.060E+000
1.632E+000
9.710E-001
1.086E+000
1.259E+000
1.336E+000
5.324E-001
1.124E+000
1.028E+000
1.207E+000
1.191E+000
8.149E-001
9.977E-001
1.011E+000
7.427E-001
7.231E-001
8.144E-001
5.763E-001
7.954E-001
1.141E+000
9.290E-001
1.324E+000
4.816E-001

NaN
