# Supplementary material for: Effects of rumen cannulation combined with different pre-weaning feeding intensities on the intestinal, splenic and thymic immune system in heifer calves several month after surgery
Source: Front Immunol. 2023 Apr 18;14:1160935. doi: 10.3389/fimmu.2023.1160935 (PMC10151785; doi:10.3389/fimmu.2023.1160935)
Supplement: Supplementary file 4 [file Table_1.docx]

Supplemental Table S1. Chemical composition and energy content (Mean ± SD) of total mixed ratio (TMR) (n= 44), starter (n = 5), hay (n = 3), and milk replacer (n = 1 pool)

| Item | TMR | Starter^1^ | Hay | Milk replacer^2^ |
| --- | --- | --- | --- | --- |
| Dry matter (%) | 43.8 ± 3.6 | 85.3 ± 0.5 | 86.4 ± 2.2 | 95.0 ± 1.3 |
| Nutrients (g/kg of dry matter) | | | | |
| Crude ash | 57.3 ± 7.3 | 78.9 ± 2.4 | 51.3 ± 2.4 | 80.3 |
| Crude protein | 159.8 ± 7.4 | 200.7 ± 7.9 | 89.0 ± 17.9 | 221.1 |
| Crude fiber | 163.8 ± 7.7 | 92.6 ± 5.7 | 315.3 ± 6.8 | < 1.0 |
| Crude fat | 31.4 ± 2.3 | 35.5 ± 1.4 | 14.0 ± 0.8 | 207.5 |
| Sugar | 14.9 ± 4.6 | 111.7 ± 7.7 | 132.6 ± 11.4 | 300.3 |
| Starch | 283.5 ± 27.4 | 292.6 ± 6.8 | n.d. | 32.3 |
| aNDF^3^ | 361.7 ± 28.3 | 222.9 ± 24.4 | 539.7 ± 7.8 | n.d. |
| ADF | 196.7 ± 14.8 | 126.4 ± 6.2 | 361.7 ± 4.9 | n.d. |
| ME (MJ/kg of DM) ^4^ | 11.3 ± 0.2 | 12.4 ± 0.3 | 8.9 ± 0.4 | 17.0 |

^1^BERGIN Kälberpellet (Bergophor Futtermittelfabrik Dr. Berger GmbH & Co. KG, Kulmbach, Germany): extracted soy meal from peeled and steam-heated beans, wheat, dried sugar beet pulp, apple pomace, apple molasses, pulped corn, wheat bran, products and by-products from bakery and pasta industry, pulped linseed, Ca(H₂PO₄)₂, CaCO3, dried yeast, dextrose. Additives per kg original substance: 16,000 IU vitamin A (rumenprotected), 4,000 IU vitamin D3, 200 mg vitamin E, 300 mg vitamin C, 4 mg vitamin K3, 15 mg vitamin B1, 8 mg vitamin B2, 10 mg vitamin B6, 50 μg vitamin B12, 50 μg vitamin B12 (rumen-protected), 20 mg Ca-Dpantothenate, 40 mg niacin amide, 4 mg folic acid, 0.3 mg biotin, 150 mg Fe, 12 mg Cu, 60 mg Zn, 50 mg Mn, 1 mg I, 0.4 mg Se. Composition: 1.2% Ca, 0.6% P, 0.3% Na.

^2^BERGIN Milch LC 50 (Bergophor Futtermittelfabrik Dr. Berger GmbH & Co. KG, Kulmbach, Germany): skimmed milk powder, whey powder, refined plant oil (palm and coconut), dextrose, egg powder. Additives per kg original substance: 25,000 IU vitamin A, 4,000 IU vitamin D3, 200 mg vitamin E, 300 mg vitamin C, 8 mg Cu, 100 mg Fe, 45 mg Zn, 30 mg Mn, 0.2 mg I, 0.2 mg Se. Composition: 1.8% Lys, 0.9% Ca, 0.7% P, 0.5% Na.

^3^aNDF = neutral detergent fiber after amylase treatment.

^4^Metabolizable energy, ME content of TMR, starter and hay was calculated according to the recommendations of the German Society of Nutrition Physiology (GfE). ME content of milk replacer was calculated following NRC (2001).

**Supplemental Table S2.** Composition and constitutes (Mean ± SD) of total mixed ratio (TMR) (n = 10)

| Ingredients (g/kg of DM) | TMR |
| --- | --- |
| Grass silage | 185.0 ± 30.9 |
| Corn silage | 471.5 ± 30.3 |
| Grass hay | 9.1 ± 12.0 |
| Straw, barley | 9.5 ± 8.4 |
| Corn, ground | 67.0 ± 2.1 |
| Wheat, ground | 16.4 ± 3.4 |
| Extracted soy meal | 18.9 ± 0.6 |
| Extracted rapeseed meal | 48.8 ± 1.5 |
| Concentrate^1^ | 160.2 ± 15.9 |
| Mineral/vitamin mix^2^ | 7.2 ± 0.2 |
| Feed supplement^3^ | 2.3 ± 3.5 |
| Lime stone powder^4^ | 2.9 ± 1.0 |
| Soy oil | 1.2 ± 0.04 |

^1^MF 2000 (Vollkraft Mischfutterwerke GmbH, Güstrow, Germany): 30% extracted soy meal from peeled and steam-heated beans, 25.4% corn grit, 15% malt culms, 6.9% extracted rapeseed meal, 5.1% wheat, 5% sugar beet pulp, 4.9% dried grain pulp, 4.1% beet molasses, 2% NaHCO3, 1% CaCO3, 0.2% NaCl. Additives per kg original substance: 10,000 IU vitamin A, 1,125 IU vitamin D3, 40 mg vitamin E, 0.6 mg I, 0.4 mg Co, 50 mg Mn, 75 mg Zn, 0.4 mg Se. Composition: 24% CP, 3.3% crude fat, 6.8% crude fiber, 8.4% crude ash, 0.7% Ca, 0.5% P, 0.65% Na.

^2^Panto-Mineral R 7609 (HL Hamburger Leistungsfutter GmbH, Hamburg, Germany): 32.8% CaNaPO3, 19.3% CaMg(CO₃)₂, 16.7% NaCl, 15.3% CaCO3, 6.1% MgO, 3% sugar cane molasses. Additives per kg original substance: 900,000 IU vitamin A, 200,000 IU vitamin D3, 4.5 g vitamin E, 1.5 g Cu, 8 g Zn, 5 g Mn, 60 mg I, 70 mg Co, 50 mg Se. Composition: 20% Ca, 6% P, 8% Na, 6% Mg.

^3^Granulate 100532 FBN (FUGEMA GmbH, Malchin, Germany): dried sugar beet pulp, extracted soy meal from peeled and steam-heated beans, rye, NaCl, NaCaPO3. Additives per kg original substance: 24,000 IU vitamin A, 4,120 IU vitamin D3, 100 mg vitamin E, 30 mg Cu, 90 mg Fe, 160 mg Zn, 100 mg Mn, 2 mg Co, 1 mg I, 1 mg Se, 14 g urea. Composition: 22.2% CP, 1.5% crude fat, 8.6% crude fiber, 8.5% crude ash, 1% Ca, 0.37% P, 0.42% Na.

^4^Kreidekalk (Spezialfutter Neuruppin GmbH und Co. KG, Neuruppin, Germany): CaCO3. Composition: 37% Ca.

Supplemental Table S3. Characteristics of primers used in the quantitative real time PCR

| Gene^1^ | Forward primer (from 5´ to 3´);  Reverse primer (from 5´ to 3´) | PCR product size [bp] | GenBank^2^ accession no. |
| --- | --- | --- | --- |
| Reference genes | | | |
| *EMD* | GCCCTCAGCTTCACTCTCAGA | 100 | NM_203361 |
|  | GAGGCGTTCCCGATCCTT |  |  |
| *LRP10* | CCAGAGGATGAGGACGATGT | 139 | Bc149232 |
|  | ATAGGGTTGCTGTCCCTGTG |  |  |
| *YWHAZ* | GAAAGGGATTGTGGACCAG | 184 | NM_174814.2 |
|  | GGCTTCATCAAATGCTGTCT |  |  |
| *Target genes* |  |  |  |
| *BOLA* | AGCTGTGGTGACTGGAGCTA | 312 | NM_001040554.1 |
|  | AGGAGTGTGCATGAGGGAAGA |  |  |
| *BOLA-DRB3* | CACAACTACGGGGGTGTGGAG | 297 | NM_001012680.2 |
|  | CTTGGGGTGATCACTTGGC |  |  |
| *CLDN1* | TTCGACTCCTTGCTGAATCTG | 84 | NM_001001854.2 |
|  | GGCTATTAGTCCCAGCAGGATG |  |  |
| *CLDN4* | CAGCGCCTTTTCAGGTCCTA | 259 | NM_001014391.2 |
|  | CGAGTCGTACACCTTGCACT |  |  |
| *FABP2* | AACTGAACTCAGTGGGGCGT | 125 | NM 001025332.1 |
|  | AAGTCTGGACCATCTCGCCA |  |  |
| *FABP6* | GGGAAACAAGAAGTTCAAGGTAC | 105 | NM 001075675.2 |
|  | ACCAGCTTGCCATCCACAAT |  |  |
| *FFAR1* | AATTCCACCAGCTCCTTGGGCAT | 213 | XM_870502 |
|  | GGCCGCCTTTAGCTTCCGTCT |  |  |
| *FFAR2* | CGCTCCTTAATTTCCTGCTG | 174 | NM_001163784 |
|  | CAAAGGACCTGCGTACGACT |  |  |
| *FFAR3* | ACCTGATGGCCCTGGTG | 215 | NM_001145233 |
|  | GGACGTGAGATAGATGGTGG |  |  |
| *FFAR4* | GGGTTCCTTTTCGATGTGAA | 166 | XM_865266 |
|  | GCCGTGACTCTTTGGAGAAG |  |  |
| *FGL2* | TTACACCGGAGCCCAGAAAC | 403 | NM_001046097.1 |
|  | TTGTCTCTATCCGGGCTGGT |  |  |
| *MUC2* | TGCAGCATCATCAAAAGCCG | 216 | XM 024987595.1 |
|  | GTCGCAGAATATGGGGCACA |  |  |
| *NOS2* | GATCCAGTGGTCGAACCTGC | 128 | NM_001076799.1 |
|  | CAGTGATGGCCGACCTGATG |  |  |
| *HCAR1* | TGC CCT TTC GGA CAG ACT AC | 218 | NM_001145234.1 |
|  | CCA AAG GAC ACA GAC AAT GC |  |  |
| *HCAR2* | GGACAGCGGGCATCATCTC | 140 | XM_015475510.1 |
|  | CCAGCGGAAGGCATCACAG |  |  |
| *IDO1* | CACCCCAAAGAAGTTTGCCG | 393 | NM 001101866.2 |
|  | TCCATGACATTGGTGCCTCC |  |  |
| *Il1O* | AACGTCCTCCGACGAGTTTC | 163 | NM_174093.1 |
|  | GCTCATGCAGAACACCACTTC |  |  |
| *Il2* | GAGTGGAACTTTCTTACCAACCAG | 222 | NM_174358.2 |
|  | TCTTCTGGCCTTGTTTTCTAGATT |  |  |
| *Il4* | GTGCTGGTCTGCTTACTGGT | 326 | NM 173921.2 |
|  | TTCAGCGTACTTGTGCTCGT |  |  |
| *Il6* | ACCCCAGGCAGACTACTTCT | 213 | NM_173923.2 |
|  | GCAAATCGCCTGATTGAACCC |  |  |
| *Il10* | CAAGGAGCACGTGAACTCACT | 92 | NM_174088.1 |
|  | TCTTGTTTTCGCAGGGCAGAA |  |  |
| *Il17A* | TCCTCCGAAGGGAGTCTCAG | 111 | NM_001008412.2 |
|  | GCTCTTAAGCCAAATGGCGG |  |  |
| *INFG* | TGTGGGCTTTTGGGTTTTTCTG | 119 | NM_174086.1 |
|  | AAGAGAGGCCCACCCTTAGC |  |  |
| *OCLN* | CCTTTTGAAAGTCCACCTCCTTAT | 70 | XM_024981258.1 |
|  | TGTCATTGCTTGGTGTGTAGT |  |  |
| *PTGES* | ACGTTCATTCTCCGTCCTCG | 292 | XM_0108051.23.1 |
|  | TCCCTCCGATCGTACCACTT |  |  |
| *PTGS1* | TTGTGGCGGACATGGCTATT | 148 | NM_001035289.3 |
|  | TGAGTGCACCTGGTCGTAAC |  |  |
| *PTGS2* | TCACTTTCATGACCACACGC | 148 | NC_006853.1 |
|  | GCGGGCAGAATGGTTCAGAT |  |  |
| *RELA* | AACAACCCCTTCCAAGTTCCC | 201 | NM_001080242.2 |
|  | CCCAGAGTTCCGATTCACCC |  |  |
| *SOD1* | AAGGCCGTGTGCGTGCTGAA | 246 | NM_174615.2 |
|  | CAGGTCTCCAACATGCCTCT |  |  |
| *TLR2* | GGT TTT AAG GCA GAA TCG TTT G | 190 | NM_174197 |
|  | AAG GCA CTG GGT TAA ACT GTG T |  |  |
| *TLR3* | TCAAAACTGCAGCACATTCCC | 93 | NM 001008664.1 |
|  | TGCCTTCACTTACTGTGACGA |  |  |
| *TLR4* | CTT GCG TAC AGG TTG TTC CTA A | 153 | NM_174198 |
|  | CTG GGA AGC TGG AGA AGT TAT G |  |  |
| *TLR6* | AGCTAAGAGCTCTCATGGCAC | 203 | NM_001001159.1 |
|  | ACTGTTAGAGCTTCCAAAATCAGC |  |  |
| *TLR9* | CTGCTGCTGTCCTACAACCA | 356 | NM 183081.1 |
|  | AAGGACAGGTTGAGTCTGCG |  |  |
| *TLR10* | CCTGAAGGCTTGACCCCAAT | 234 | NM 001076918.2 |
|  | TCT GAG ACC TGC CAG TGA GA |  |  |
| *TGFB1* | CCGAACCTGTGTTGCTCTCT | 419 | NM 001166068.1 |
|  | GTAGTTGGTGTCCAGGGCTC |  |  |
| *TNFA* | AGAGGGAAGAGCAGTCCCCAG | 181 | NM_173966.3 |
|  | TTCACACCGTTGGCCATGAG |  |  |
| *ZO1* | AGAAAGATGTTTATCGTCGCATCGT | 84 | XM_024982012.1 |
|  | ATTCCTTCTCATATTCAAAATGGGTTCTGA |  |  |
| *ZO2* | TGCTCCATTCATTTGCGGTTC | 70 | XM_024995499.1 |
|  | GGCCTCTTGACCACAATGG |  |  |

^1^*EMD*, emerin; *LRP10*, LDL receptor related protein 10; *YWHAZ*, tyrosine 3-monooxygnase/tryptophan 5-monooxygenase activation protein zeta; *BOLA*, major histocompatibility complex (MHC), class I; *BOLA-DRB3*, MHC class II; *CLDN1*, Claudin 1; *CLDN4*, Claudin 4; *FABP2*, fatty acid binding protein 2; *FABP6*, fatty acid binding protein 6; *FFAR*, free fatty acid receptor; *FGL2*, fibrinogen-like protein 2; *MUC2*, mucin2; *NOS2*, nitric oxide synthase 2; *HCAR1*, hydroxycarboxylic acid receptor 1; *HCAR2*, hydroxycarboxylic acid receptor 2 ;*IDO1* , indoleamine 2,3 diogygenase; *IL1B*, interleukin 1β; *IL2*, interleukin 2; *IL4*, interleukin 4; *IL6*, interleukin 6; *IL10*, interleukin 10, *IL17A*, interleukin 17α; *INFG*, interferon γ; *OCLN*, occluding; *PTGES*, prostaglandin endoperoxide synthase; *PTGES1*; prostaglandin endoperoxide synthase 1 (Cox 1); *PTGES2*, prostaglandin endoperoxide synthase 2 (Cox 2); *RELA*, Rela proto-oncogene/NF-κB subunit; *SOD1*, superoxide dismutase 1; *TLR*, toll-like receptor; *TGFB1*, transforming growth factor β 1; *TNFA*, tumor necrosis factor α; *ZO1*, tight junction protein 1 (TJP1); *ZO2*, tight junction protein 2 (TJP2)

^2^Database used: National Center for Biotechnology Information (NCBI) Entrez Nucleotide (<http://www.ncbi.nlm.nih.gov/nucleotide>)
